# Regulation of Podocyte Injury by CircHIPK3/FUS Complex in Diabetic Kidney Disease

**DOI:** 10.7150/ijbs.75994

**Published:** 2022-09-01

**Authors:** Feng Liu, Jing Huang, Chunyun Zhang, Yaru Xie, Yiling Cao, Li Tao, Hui Tang, Jihong Lin, Hans-Peter Hammes, Kun Huang, Fan Yi, Hua Su, Chun Zhang

**Affiliations:** 1 Department of Nephrology, Union Hospital, Tongji Medical College, Huazhong University of Science and Technology, Wuhan 430022, China.; 2 5th Medical Department, Medical Faculty Mannheim, University of Heidelberg, D-68167 Mannheim, Germany.; 3 Tongji School of Pharmacy, Tongji Medical College, Huazhong University of science and Technology, Wuhan 430030, China.; 4 Department of Pharmacology, School of Basic Medical Sciences, Shandong University, Jinan 250012, China.

**Keywords:** podocyte injury, diabetic kidney diseases, circHIPK3, FUS, EDA2R

## Abstract

Diabetic kidney disease (DKD) is a major microvascular complication of diabetes mellitus and is one of the leading causes of end-stage kidney disease. Circular RNAs (circRNAs) are a class of endogenous non-coding RNAs that play important roles in various diseases, yet their roles in DKD are poorly understood. CircRNA HIPK3 (circHIPK3), a highly conserved circRNA, is closely related to various cellular functions, including cell proliferation and apoptosis. The association between circHIPK3 and diabetic complications has been well demonstrated in multiple previous studies. However, the role of circHIPK3 in podocyte injury in DKD remains unclear. Herein, we discovered that circHIPK3 expression is markedly elevated in cultured podocytes under high-glucose (HG) conditions and glomeruli of diabetic mice, which is closely associated with podocyte injury in DKD. Functionally, lentivirus-mediated knockdown of circHIPK3 dramatically suppresses HG-induced podocyte apoptosis* in vitro*. Therapeutically, silencing circHIPK3 by adeno-associated virus-mediated RNA interference ameliorates podocyte injury and albuminuria in STZ-induced diabetic mice. Mechanistically, circHIPK3 facilitates the enrichment of fused in sarcoma (FUS) on the ectodysplasin A2 receptor (EDA2R) promoter, resulting in the upregulation of EDA2R expression and activation of apoptotic signaling. Taken together, these results indicate circHIPK3/FUS/EDA2R axis as a therapeutic target for podocyte injury and DKD progression.

## Introduction

Diabetic kidney disease (DKD), occurring in ~40% of patients with diabetes, is one of the most common and devastating diabetic microvascular complications, which leads to impaired renal function 1, 2. Although current treatment strategies (intensive glucose control and anti-hypertensive therapies) have meaningfully improved outcomes for diabetes complications, DKD still poses a major risk factor for the progression towards end-stage kidney disease 1. Abundant evidence indicates that podocyte injury is a critical risk factor for progression of DKD, leading to proteinuria and further kidney damage 3, 4. Thus, identifying key molecules involved in podocyte injury is of great importance for the development of novel therapeutic strategies for the treatment of patients with DKD. However, to date, the precise molecular mechanisms contributing to podocyte injury and development of DKD have yet to be elucidated. Therefore, effective therapeutic strategies aimed at attenuating or preventing podocyte injury exert the potential to bring huge clinical benefits and economic profits to global health systems.

Previous studies have demonstrated that non-coding RNAs (ncRNAs), such as Tug1 5, 6, miR-93 7, 8, and miR-21 8, 9, contribute to the development and progression of podocyte injury in DKD. CircRNAs are a novel class of endogenous expressed ncRNAs with covalently closed structure and single-stranded that arise from exons or introns, which can regulate gene expression by acting as miRNA sponges, transcription regulators, or RNA-binding protein partners 10, 11. Previous studies have demonstrated that dysregulated expression of circRNAs is associated with multiple complicated diseases 10-13, including diabetic microvascular complications. For example, circular RNA HIPK3 (circHIPK3) 14, circular RNA PWWP2A (cPWWP2A) 15, and circular RNA ZNF532 (circRNA-ZNF532) 16 have been discovered to play important roles in the development of diabetes-induced retinal vascular leakage. Notably, it has been shown that circRNA.4614 can be considered as potential therapeutic target for diabetic neuropathic pain 17. Likewise, other studies have also demonstrated that circRNA_010383 18, and circ_DLGAP4 19 play critical roles in the initiation and progression of renal fibrosis in DKD. However, to date, little research has been conducted on the potential implications of circRNAs in diabetic podocyte injury. Therefore, more dysregulated circRNAs need to be identified for exploring molecular mechanisms associated with podocyte injury in DKD progression, which is anticipated to make a potential breakthrough in the clinical application for the treatment of DKD.

CircHIPK3, a highly conserved circRNA across different species, is generated from the circularization of HIPK3 gene exon 2, which is expressed ubiquitously in mammalian tissues. Emerging studies have demonstrated that dysregulation of circHIPK3 is associated (perhaps causally) with numerous disease states 20, including multiple diabetes-related complications. However, so far, rare reports have addressed the role of circHIPK3 in the pathogenesis of podocyte injury under diabetic conditions along with its implication on DKD progression. In this study, we identify circHIPK3 upregulation as a key driver of podocyte injury under diabetic conditions, which can promote podocyte loss and DKD progression. Mechanistically, circHIPK3 facilitates the enrichment of fused in sarcoma (FUS) on the ectodysplasin A2 receptor (EDA2R) promoter, resulting in the upregulation of EDA2R expression and activation of apoptotic signaling. *In vivo* administration of adeno-associated virus-mediated short hairpin RNA (AAV-shRNA) targeting circHIPK3 significantly ameliorates podocyte injury and decreases proteinuria in diabetic mice, indicating the pivotal roles of circHIPK3/FUS/EDA2R axis in DKD progression.

## Methods

### Cell culture

The conditionally immortalized human podocyte cell line (HPC) was kindly provided by Dr. Heping Ma (Emory University) and the conditionally immortalized mouse podocyte cell line (MPC-5) was obtained from American Type Culture Collection. HPC cells were cultured in RPMI 1640 medium supplemented with 10% fetal bovine serum (FBS; 10099-141, Gibco, Australia) and MPC-5 cells were propagated in DMEM medium supplemented with 10% FBS as previously described 21, 22. All cell lines have been authenticated by STR profiling and tested negative for mycoplasma contamination during the period of this study. When the podocytes reached approximately 70%-80% confluence, the cells were maintained at 37 °C for 10-14 days to induce differentiation and then were used for subsequent experiments. Hsa_circ_0000284 overexpression lentivirus and an empty lentivirus, as well as hsa_circ_0000284/mmu_circ_0001052 shRNA lentiviral particle and the indicated scramble shRNA lentiviral were obtained from GeneChem (Shanghai, China). Podocytes were transfected with lentivirus to overexpress or interfere circHIPK3 expression according to the manufacturer's instructions.

### RNA isolation and quantitative Real Time PCR (qRT-PCR)

Total RNAs were isolated from cultured cells and mouse glomeruli with FastPure Cell/Tissue Total RNA Isolation Kit (RC112-01, Vazyme, Nanjing) according to directions of the manufacturer. qRT-PCR (Q311-02, Vazyme, Nanjing) was conducted with the StepOne Plus qRT-PCR System (Thermo Fischer Scientific). The specific primers sequences used in this study are listed in Supplementary Table 1.

### RNase R and actinomycin D treatment

RNase R (R7092M, Beyotime, China) and actinomycin D (HY-17559, MCE, China) treatment assays were conducted as previously illustrated 14, 18.

### RNA fluorescence in situ hybridization (RNA-FISH)

Cy3-labeled circHIPK3, 18s, and U6 probes were acquired from RiboBio (Guangzhou, China). The signal intensity of cy3-labeled probes was detected by the Fluorescent in situ Hybridization Kit (C10910, RiboBio, China) according to directions of the manufacturer. All fluorescence images were captured using Nikon-A1Si Laser Scanning Confocal Microscope (Nikon, Japan).

### Nuclear and cytoplasmic extraction

Subcellular fractionation of HPC cells was performed using Cytoplasmic & Nuclear RNA Purification Kit (21000, Norgen Biotek, Canada). Total RNAs were subsequently extracted from both nuclear and cytoplasmic fractions. Then equal volumes of RNA from both fractions were subjected to qRT-PCR (Q311-02, Vazyme, Nanjing) analysis.

### RNA pull-down

The biotinylated DNA probes-binding complex was precipitated by incubating streptavidin-coated magnetic beads (M-280, Invitrogen) with cell lysates according to the manufacturer's operating instructions. The relative circHIPK3 enrichment in the sediment was detected by qRT-PCR assay. The binding proteins were subsequently eluted and separated by SDS-PAGE gel (SW109-01, Sevenbio, Beijing, China) electrophoresis, and followed by Coomassie blue staining (P1305, Solarbio, Beijing) or Western blot.

### Immunoblot analysis

Total protein extracted from cultured cells or mouse glomeruli was subjected to electrophoresis on SDS-PAGE gels (Sevenbio, Beijing, China) and transferred to polyvinylidene fluoride (PVDF) membranes (Millipore, Missouri, USA). After blocking, the membranes were probed with primary and the corresponding secondary antibodies. Then, immunoreactive bands were visualized by Omni-ECL Enhanced Pico Light Chemiluminescence kit (SQ101, Epizyme Biotech, China). Primary antibodies included: Desmin (ab32362, abcam), IGF2BP1 (ab184305, abcam), IGF2BP3 (ab225697, abcam), CORO1A (ab203698, abcam), BAX (50599-2-Ig, Proteintech), DYKDDDDK Tag (20543-1-AP, Proteintech), IGF2BP2 (11601-1-AP, Proteintech), FUS (11570-1-AP, Proteintech), Cleaved Caspase-3 (Asp175, R&D), Polyclonal anti-podocin (p0372, Sigma-Aldrich), and EDA2R (sc-377423, Santa).

### Cross-linking RNA immunoprecipitation (RIP)

Cultured cells were cross-linked at 200 mJ/cm^2^ using 254 nm UV light with an Ultraviolet Crosslinker (UVP) before being lysed as previously described 23. RIP assays were conducted using the Magna RIP Kit (17-700, Millipore, MA), with antibodies specific for FUS (11570-1-AP, Proteintech), IGF2BP1 (ab184305, abcam), IGF2BP2 (11601-1-AP, Proteintech), IGF2BP3 (ab225697, abcam), or DYKDDDDK Tag (20543-1-AP, Proteintech). The precipitated RNAs were then analyzed by qRT-PCR. The relative amount of immunoprecipitated RNA in each sample was represented as that of negative (IgG) sample.

### *In vitro* binding assay

A series of FUS truncates were amplified with primer sets (Supplementary Table 1) and subcloned into pCMV 3xFLAG plasmid (Addgene). The FUS-circRNA complexes were pulled down using Anti-Flag magnetic beads (HY-K0207, MCE, China). Protein was measured by Western blot and circRNA expression was detected by qRT-PCR with specific divergent primers (Supplementary Table 1).

### Chromatin immunoprecipitation (ChIP)

ChIP assay was conducted using the ChIP Assay kit (P2078, Beyotime, China) according to the manual instruction. Chromatin samples were immunoprecipitated with antibodies against FUS (11570-1-AP, Proteintech) or a negative control Rabbit IgG antibody (31235, Thermo Fisher Scientific). After purification of DNA, gene promoter specific sequences were amplified by qRT-PCR with specific primers. Data were calculated based on fold enrichment of FUS pull-down versus IgG pull-down.

### RNA sequencing (RNA-Seq)

RNA sequencing (RNA-seq) experiments were conducted by Seqhealth Technology Co. (Wuhan, China). Briefly, total RNAs were extracted from duplicate of HPC cells using the Total RNA Isolation Kit (Vazyme). Differentially expressed mRNAs between groups were identified with an adjust *P*-value cutoff of 0.05 and an inter-group absolute fold-change cutoff of 1.5. Differential gene expression information is listed in Supplementary Table 3. Raw data were deposited in the Gene Expression Omnibus database (GSE194107).

### Flow cytometric analysis

Podocyte apoptosis rates were examined using a commercial Annexin V-PE/7-AAD Apoptosis Detection Kit (A213-02, Vazyme, Nanjing) according to the manufacturer's instruction and analyzed by flow cytometer (BD Bioscience).

### Dual-luciferase reporter assay

The wild-type and the FUS binding site (-569/-562) mutation of human EDA2R promoter (-2,000/-1) were synthesized by AuGCT DNA-SYN Biotechnology Co. Ltd. (Beijing, China) and inserted into the upstream of the firefly luciferase gene in pGL3-Basic luciferase reporter vector (Promega, USA). Luciferase activities were detected by using the Dual-Luciferase® Reporter Assay System kit (E1910, Promega, USA) according to the manufacturer's protocol.

### Human renal biopsy samples

Kidney biopsy samples from patients with biopsy-proven DKD were collected at Union Hospital of Tongji Medical College of Huazhong University of Science and Technology (HUST). DKD diagnosis was verified by kidney histological and transmission electron microscopy analysis. Renal samples from patients who received radical nephrectomy for renal carcinoma and without diabetes or kidney disease were collected as control. All specimens were obtained with appropriate informed consent from the patients and approved by the Institutional Review Board of Tongji Medical College of HUST.

### Animal studies

All animals were kept in the Specific Pathogen Free (SPF) Laboratory Animal Center of Huazhong University of Science and Technology according to NIH Guidelines for the Care and Use of Laboratory Animals (Approval number: [2021] 2611). Eight-week-old C57BL/6J male mice (GemPharmatech Co.) were treated with an intraperitoneal injection of streptozotocin (150 mg/kg) 24, 25. One week after streptozotocin injection, mice with blood glucose >16.7 mmol/L were included in the experiments. The recombinant AAV carrying a shRNA against mmu_circ_0001052 (AAV-shRNA) or a negative control shRNA (AAV-scram) were constructed by OBiO Technology (Shanghai, China). After two months of establishment of the DKD model, AAV (2^10^11^ pfu/per mouse) were delivered by tail vein injection 26, 27. All mice were killed at 12 weeks after the first injection of AAV. Blood, urine, and kidney tissue specimens were collected for histological and biochemical analyses.

### Glomeruli isolation

Mouse glomeruli were isolated according to our previously described 25, 28. Briefly, mice were anesthetized and the distal abdominal aorta and inferior vena cava, superior mesenteric artery and celiac artery were surgically ligated. After kidneys were perfused with 4^10^7^ inactivated Dynabeads (M-450, Invitrogen) diluted in 10 mL Hank's Balanced Salt Solution (preheated at 37 °C), kidneys were removed immediately and cut into small pieces on ice. Then the minced kidney tissue was digested at 37 °C for 15 mins in a corresponding digestion buffer (consist of 50 U/ml DNase I, 300 U/ml collagenase type II, 1 mg/ml proteinase E). The digested tissue was subsequently lightly filtered through a 100 μm cell strainer, followed by centrifugation at 1500 rpm. After strict washing, the isolated glomeruli were used for RNA isolation and subsequent qRT-PCR analysis as well as protein extraction and Western blot analysis.

### Transmission electron microscopy analysis

The handling and examination of electron microscopic samples (mouse kidneys) were performed by the Wuhan Institute of Virology (WIV) of the Chinese Academy of Science (CAS) as described in our previous study 29.

### Statistics

All the data statistical analyses in this study were implemented by using GraphPad Prism 8.0 software (La Jolla, USA) and data were expressed as mean ± SD from at least three independent experiments. Unpaired Student's t tests were used to analyze the difference between two groups. One-way ANOVA test was used for comparisons among multiple groups. Spearman's correlation analysis was used to analyze the correlation between different two genes. All statistical analyses were two-tailed, and a *P* value of <0.05 was considered statistically significant.

## Results

### CircHIPK3 is up-regulated in HG-stimulated podocyte and participates in HG-induced podocyte apoptosis

**Figure 1:**
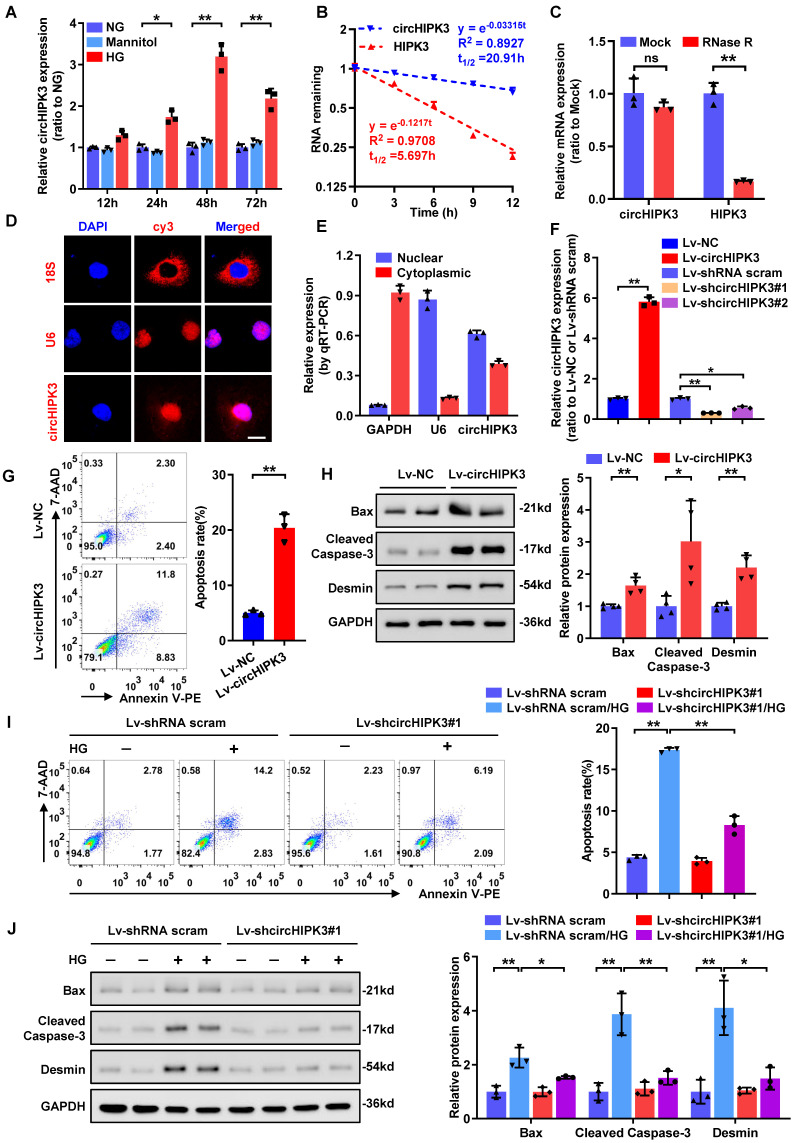
** CircHIPK3 is up-regulated in HG-stimulated podocyte and participates in HG-induced podocyte injury. A** Summarized data of qRT-PCR showing the relative levels of circHIPK3 in HPC treated with 5.5 mmol/L glucose (NG), 40 mmol/L glucose (HG), or mannitol (34.5 mmol/L mannitol plus 5.5 mmol/L glucose) conditions for indicated time. **B** qRT-PCR assays were conducted to detect the amount of circHIPK3 and HIPK3 mRNA in podocytes after actinomycin D treatment. **C** Total RNAs were digested with RNase R followed by qRT-PCR detection of circHIPK3 and HIPK3 mRNA expression levels. **D** Identification of circHIPK3 cytoplasmic and nuclear distribution by RNA FISH in HPC using a cy3-labled junction specific antisense probe (red), with the nuclei staining with DAPI (blue). The 18S and U6 were applied as positive controls. Scale bar, 10 μm. **E** Identification of circHIPK3 cytoplasmic and nuclear distribution by qRT-PCR analysis in HPC. GAPDH and U6 were used as cytoplasm and nuclear control, respectively. **F** Validation of lentivirus-mediated circHIPK3 overexpression and knockdown efficiencies by qRT-PCR analysis in HPC cells. **G** HPC cells with different treatments were stained with 7-AAD and Annexin V-PE, and analyzed by flow cytometry to evaluate the apoptosis rate. Quantification of the apoptotic cells was showed at right panel (n=3). **H** Representative Western blot gel images (left panel) and summarized data (right panel) showing the relative protein levels of Cleaved Caspase-3, Bax, as well as Desmin in podocytes with different treatments. GAPDH served as loading control (n=4). **I** The effect of circHIPK3 knockdown on HG-induced HPC apoptosis and the quantification data (n=3). **J** Representative Western blot gel images (left panel) and summarized data showing the relative protein levels of the indicated proteins in HPC with different treatments. GAPDH served as loading control (n=3). Data are expressed as mean ± SD of three or four independent experiments. One-way ANOVA was used for comparison among multiple groups. Student's t-test was employed for comparisons between two groups. *P < 0.05; **P < 0.01; ns means no significant.

Prior studies have shown that circHIPK3 expression is significantly up-regulated in high-glucose (HG)-treated human retinal endothelial cells and in diabetic mouse retinas 14, implying a potential role of circHIPK3 in diabetic microvascular complications. However, it still remains to be elusive whether circHIPK3 is implicated in podocyte injury in DKD. To determine whether circHIPK3 is involved in HG-induced podocyte injury, we firstly examined the expression pattern of circHIPK3 in HPC cells after HG treatment. qRT-PCR analysis indicated that HG treatment dramatically increased the expression level of circHIPK3 in HPC (Fig. 1A), indicating that circHIPK3 may play an essential role in HG‐induced podocyte injury. Resistant to actinomycin D treatment and RNase R exonuclease digestion confirmed that circHIPK3 exhibited high stability in HPC (Fig. 1B-C). In addition, RNA-FISH detection (Fig. 1D) and qRT-PCR analysis of nuclear and cytoplasmic RNA fractions (Fig. 1E) showed that a large part of circHIPK3 was localized in the nucleus of HPC.

To explore the possible biological function of circHIPK3 in podocytes, lentivirus-mediated circHIPK3 overexpression and knockdown in HPC cells were employed (Fig. 1F). Functional studies revealed that ectopic expression of circHIPK3 resulted in increased podocyte apoptosis (Fig. 1G) and up-regulated expression of pro-apoptosis proteins (Fig. 1H), mimicking the effect of HG stimulation. In contrast, silencing of circHIPK3 decreased cell apoptosis in HG-treated podocyte (Fig. 1I). However, circHIPK3 knockdown did not have a significant effect under normal-glucose conditions (Fig. 1I). These results were further confirmed at protein levels by Western blotting assay (Fig. 1J). Collectively, these findings indicated that circHIPK3 was highly expressed in HG-stimulated podocytes and promoted podocyte injury under diabetic conditions.

### CircHIPK3 interacts with FUS protein in human podocyte

**Figure 2:**
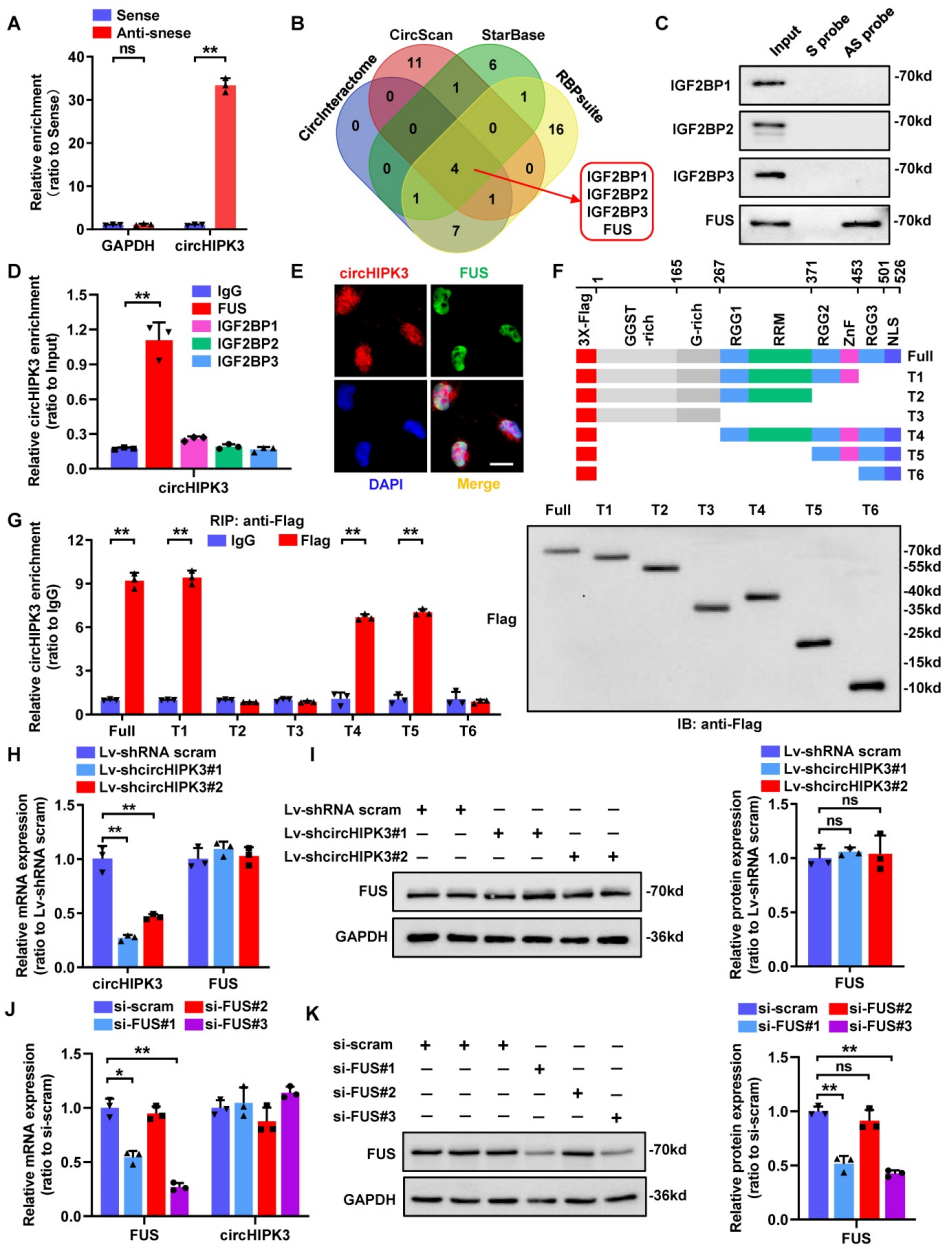
** CircHIPK3 could interact with FUS in podocyte nucleus. A** HPC lysates were subjected to RNA pull-down assay. The expression of circHIPK3 was tested by qRT-PCR. **B** Bioinformatics analysis indicates that IGF2BP1, IGF2BP2, IGF2BP3 and FUS are likely to interact with circHIPK3. **C** Biotin-labeled RNA pull-down and Western blot assays showing protein pulled down by circHIPK3 from HPC lysates. **D** RIP assays using anti-IGF2BP1, IGF2BP2, IGF2BP3, FUS or IgG control antibodies and qRT-PCR assays showing the interaction between circHIPK3 and above proteins in HPC cells. **E** Dual RNA-FISH and immunofluorescence staining assay indicating the colocalization of circHIPK3 (red) and FUS (green) in HPC cells. **F** Schematic diagram revealing the domains of FUS truncations. **G** *In vitro* binding assay depicting the recovered circHIPK3 levels detected by qRT-PCR (left panel) after incubation with full-length or truncation forms of Flag-tagged recombinant FUS protein validated by Western blot (right panel). **H, I** Expression of FUS and circHIPK3 was confirmed by qRT-PCR (H) or Western blot (I) in HPC transfected with Lv-circHIPK3 shRNAs (n=3). **J, K** Expression of circHIPK3 and FUS were verified by qRT-PCR (J) or Western blot (K) in HPC transfected with FUS siRNAs (n=3). Data are shown as mean ± SD of three independent experiments. Student's t-test was employed for comparisons between two groups. *P < 0.05, **P < 0.01; ns represents no significant.

Besides their function as miRNA sponges, circRNAs can exert their biological functions by interacting with RNA-binding proteins 30. Given that large part of circHIPK3 locates in podocyte nucleus, we next conducted RNA pull-down assay to investigate its protein binding role (Fig. 2A). Then the precipitates were separated by SDS-PAGE followed by Coomassie bright blue staining, and a specific band of ~70 kD was detected in anti-sense probe extracts (Supplementary Fig. 1A). To further explore the potential binding proteins, we performed an in-silico analysis using four bioinformatic website tools (CircInteractome, CircScan, RBPsuite, and StarBase) to predict proteins that may bind to circHIPK3. Among the candidate proteins, four proteins (IGF2BP1/2/3, and FUS) were predicted by all four databases (Fig. 2B; Supplementary Table 2), which was further identified to be FUS by RNA pull-down assay followed by Western blotting (Fig. 2C) and RNA immunoprecipitation (RIP) assay (Supplementary Fig. 1B; Fig. 2D) verification. We further confirmed the co-localization of endogenously expressed circHIPK3 and FUS in the nucleus of podocyte by performing RNA-FISH combined with immunofluorescence (Fig. 2E).

Studies have shown that the C-terminal region of FUS protein, consisting of an RNA recognition motif (RRM) and a zinc finger (ZnF) domain, is an RNA binding motif 31. To explore whether the two globular domains of FUS are essential for interactions between FUS and circHIPK3, we constructed a series of FUS truncations (Fig. 2F).* In vitro* binding assays showed that deletion of ZnF (372-453 amino acids), but not the RRM (268-371 amino acids), significantly impaired the interaction between FUS and circHIPK3 (Fig. 2G), suggesting that the ZnF domain was the key region involved in the circHIPK3-FUS interaction. In addition, knockdown of circHIPK3 did not affect FUS expression (Fig. 2H-I), nor did silencing FUS alter the expression level of circHIPK3 (Fig. 2J-K), indicating that their expression levels were independently regulated in HPC. Taken together, our results proposed that circHIPK3/FUS formed an RNA-protein complex through the ZnF domain of FUS in HPC.

### CircHIPK3 promotes podocyte damage by upregulating EDA2R

**Figure 3:**
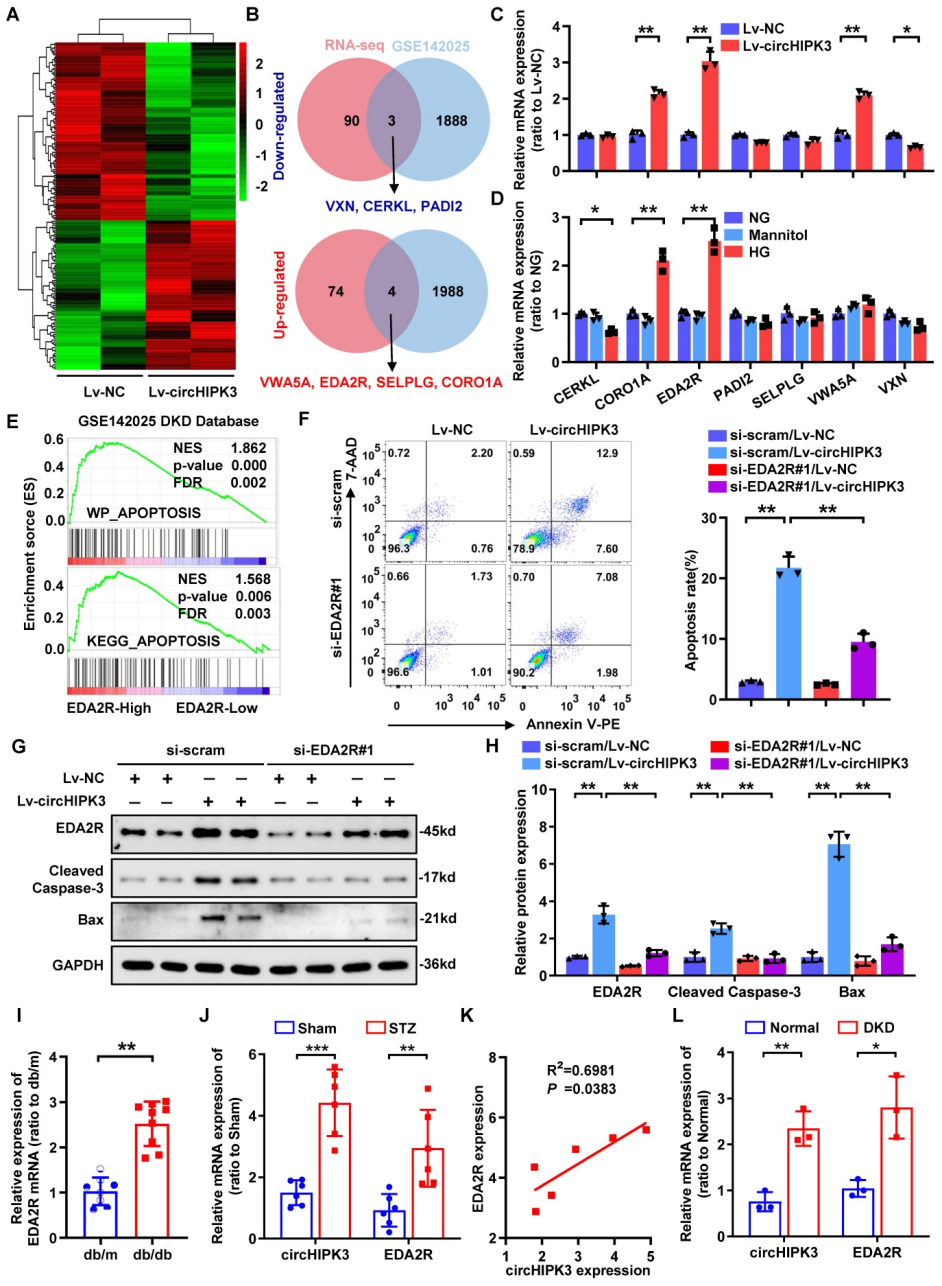
** CircHIPK3 promotes podocyte damage by upregulating EDA2R. A** Clustered heatmap of significant differentially expressed mRNAs in HPC transfected with circHIPK3 overexpression lentivirus versus control lentivirus. Each sample contained a mixture of three repeats. **B** Schematic flowchart showed the overlapping of circHIPK3-regulated mRNAs identified by RNA-seq in HPC cells and published GSE142025 dataset in human DKD (filtered by fold change > 1.5 or < -1.5 and adjust p-value < 0.05). **C** Expression levels of indicated mRNAs in HPC cells with circHIPK3 overexpression. **D** Expression levels of indicated mRNAs in HPC cells under HG (40 mmol/L) stimulation. **E** Gene set enrichment analysis (GSEA) of GEO datasets (GSE142025) showing that higher EDA2R expression is significantly associated with apoptosis in DKD. **F** HPC cells with different treatments were stained with Annexin V-PE and 7-AAD, and analyzed by flow cytometry to evaluate cell apoptosis rate. Summarized was showed at right panel (n = 3). **G, H** Western blot analysis of EDA2R, Bax, and Cleaved Caspase-3 in HPC with different treatments (n=3). **I** Nephroseq expression data for EDA2R in db/m mice glomeruli (n=8) and db/db mice glomeruli (n=9).** J** qRT-PCR assays were conducted to detect circHIPK3 and EDA2R expression in STZ-induced diabetic mouse glomeruli (n=6) after five months diabetes induction, in comparison with the control mouse glomeruli (n=6). Expression values were normalized to ACTB mRNA. **K** Correlation analysis revealed positive correlation between the levels of circHIPK3 and EDA2R mRNA from the diabetic mouse glomeruli. **L** qRT-PCR analysis of circHIPK3 and EDA2R expression levels in renal biopsy samples from patients with DKD (n = 3 for control, n = 3 for patients with DKD). Data are shown as mean ± SD of three independent experiments. Student's t-test was employed for comparisons between two groups. One-way ANOVA was used for comparison among multiple groups. Correlation between circHIPK3 and EDA2R expression was analyzed by Pearson's correlation. *P < 0.05, **P < 0.01, ***P < 0.001.

Next, we analyzed potential downstream targets of the increased expression of circHIPK3 accounting for HG-induced podocyte injury. RNA-seq assay revealed 78 up-regulated and 93 down-regulated genes (Fold change > 1.5 or < -1.5, and adjust *P*-value < 0.05) in HPC upon circHIPK3 overexpression (Fig. 3A; Supplementary Table 3). To identify essential regulators in podocyte injury associated with DKD, we performed a comprehensive analysis of the public DKD datasets (GSE142025) and identified 1992 up-regulated and 1891 down-regulated genes in DKD specimens. Combined analysis of our RNA-seq data and GSE142025, we finally identified 7 differentially expressed genes that are likely to be associated with podocyte injury in DKD (Fig. 3B), but only 4 genes were significantly different in subsequent qRT-PCR verification (Fig. 3C). We speculate that this inconsistency is caused by the low expression abundance of the gene itself, or it may be caused by the interference of homologous gene expression 32, 33. Interestingly, the expression of ectodysplasin A2 receptor (EDA2R) and coronin 1A (CORO1A) were consistently upregulated upon circHIPK3 overexpression or HG stimulation (Fig. 3C-D; Supplementary Fig. 2A-B). Of note, gene set enrichment analysis (GSEA) indicated that EDA2R (Fig. 3E), but not CORO1A (Supplementary Fig. 2C), was highly correlated with apoptosis-associated pathways based on the data from GSE142025. To further confirm whether circHIPK3-induced podocyte injury via EDA2R or CORO1A, we conducted a series of rescue experiments. The results showed that circHIPK3 induced increased podocytes apoptosis were significantly diminished only in EDA2R-deficient podocyte (Supplementary Fig. 2D-F; Fig. 3F), suggesting that EDA2R mediated circHIPK3-induced podocyte injury. Moreover, EDA2R silencing reversed Caspase-3 activation and EDA2R upregulation in circHIPK3-overexpressed podocytes (Fig. 3G-H). Hence, EDA2R is considered as an important candidate target of circHIPK3.

In addition, EDA2R was significantly increased in micro-dissected glomeruli from subjects with db/db mice based on a publicly available data set from Nephroseq (https://www.nephroseq.org/) (Fig. 3I). From the same database, higher expression levels of EDA2R were positively correlated with increased levels of urinary albumin/creatinine ratio (UACR) in db/db mice (Supplementary Fig. 2G). Consistently, our animal studies indicated that EDA2R expression was significant up-regulated in glomeruli from STZ-induced diabetic mice as compared with non-diabetic mice (Fig. 3J; Supplementary Fig. 2H-I). Meanwhile, analysis of the correlation between EDA2R and circHIPK3 transcriptional levels showed that EDA2R was positively correlated with circHIPK3 expression in glomeruli micro-dissected from diabetic kidneys (Fig. 3K). These data were confirmed in renal biopsy samples from patients with biopsy-proven DKD (Fig. 3L). From these findings, we concluded that circHIPK3-induced podocyte injury probably mainly through upregulating EDA2R expression.

### CircHIPK3 facilitates the expression of EDA2R through FUS

**Figure 4:**
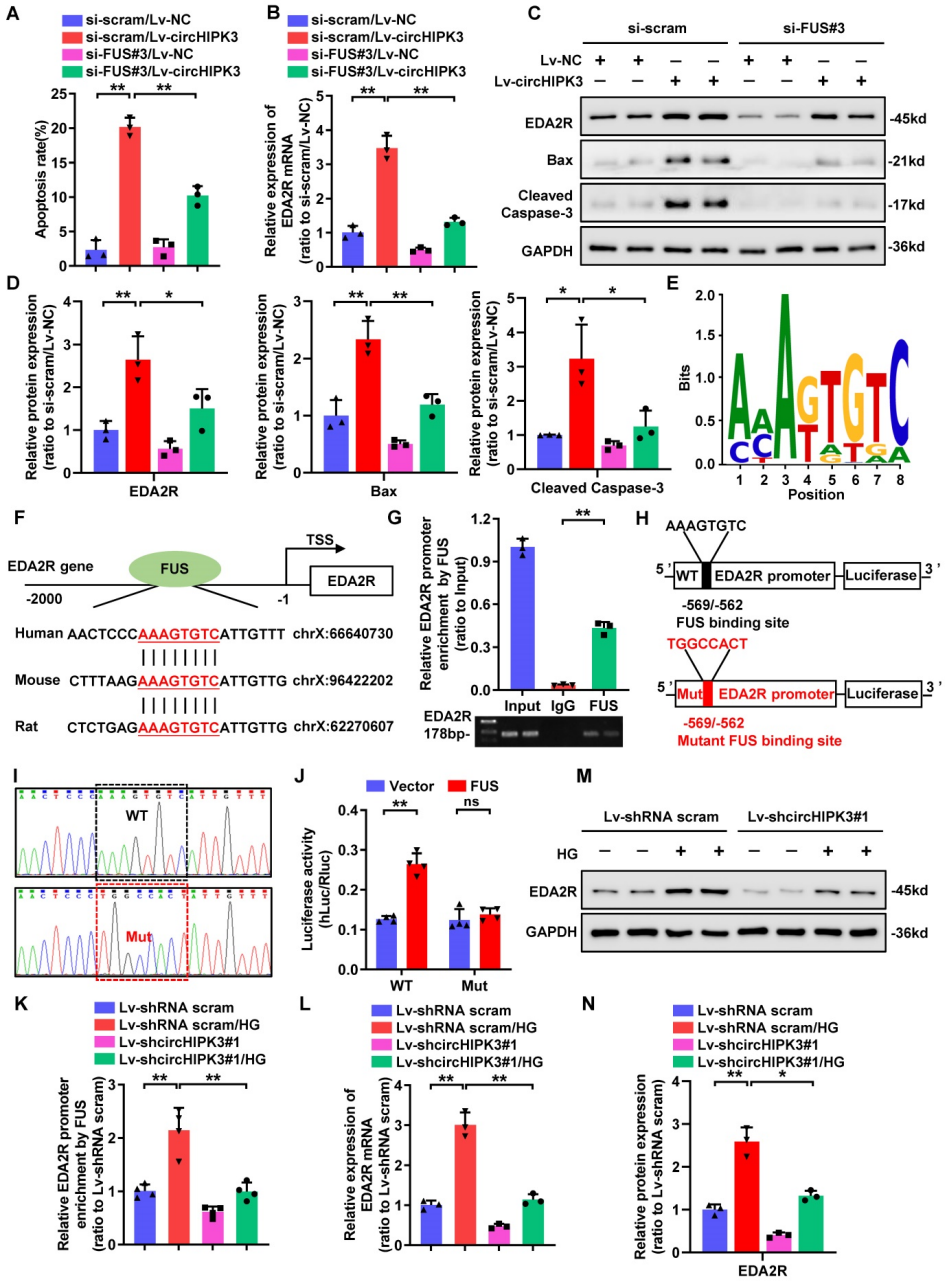
** CircHIPK3 facilitates the expression of EDA2R through FUS. A** Summarized data showing the level of cell apoptosis determined by flow cytometric analysis in podocytes with different treatments (n=3). **B** qRT-PCR analysis of EDA2R mRNA expression levels in HPC cells transfected with Lv-NC or Lv-circHIPK3 and those co-transfected with si-FUS#3 or si-scram. **C, D** Western blot analysis of EDA2R, Bax, and Cleaved Caspase-3 in HPC with different treatments (n=3). **E** The predicted sequence of FUS-binding motif as identified by MatrixREDUCE. **F** The predicted FUS binding site at the promoter of the evolutionarily conserved region of EDA2R in human, mouse and rat, indicated by red and underlined. **G** Chromatin immunoprecipitation (ChIP) assays using FUS antibody indicating that FUS could bind to promoter of EDA2R gene in HPC cells. IgG was applied as negative control. **H, I** Schematic model of mutation (H) and sequencing of mutation (I). **J** Luciferase activity of the reporter vector containing the wide type (WT) or mutant (Mut) promoter of EDA2R was determined after co-transfection with control or FUS expressing plasmids in HPC cells. **K** ChIP analysis showed that the interaction between EDA2R gene promoter and FUS protein in HPC cells with different treatments. **L** qRT-PCR analysis of EDA2R mRNA expression levels in HPC cells with indicated treatments. **M**,** N** Representative Western blot gel images (M) and summarized data (N) showing the effect of circHIPK3 knockdown on the protein level of EDA2R in HPC cells treated with HG (40mmol/L) (n=3). Values are expressed as mean ± SD of three or four independent experiments. One-way ANOVA was used for comparison among multiple groups. Student's t-test was employed for comparisons between two groups. *P < 0.05, **P < 0.01; ns represents no significant.

We then returned to consider how circHIPK3 regulated the expression of EDA2R. Previous studies have confirmed that circRNAs located in the nucleus are mainly involved in transcriptional regulation through interacting with DNA-binding proteins or recruiting transcription cofactors 11-13. To investigate the role of circHIPK3/FUS complex in regulating EDA2R expression, we first performed FUS knockdown in circHIPK3-overexpressed HPC. Flow cytometric analysis showed that silencing of FUS significantly inhibited circHIPK3-induced HPC apoptosis (Fig. 4A). Furthermore, knockdown of FUS suppressed circHIPK3-induced upregulation of EDA2R at both mRNA and protein levels (Fig. 4B-C), as well as increased Cleaved Caspase-3 and Bax protein expression (Fig. 4C-D), suggesting that circHIPK3-induced podocytes apoptosis and upregulation of EDA2R were largely dependent on FUS. Considering that FUS could regulate transcription in the nucleus 34, 35, we speculated that circHIPK3 may regulate EDA2R expression through interacting with FUS at transcription level. To verify this hypothesis, we scanned the DNA sequence of EDA2R promoter for putative FUS binding sites. Interestingly, one highly conserved putative binding site for FUS was identified in EDA2R promoter DNA sequence (Fig. 4E-F). To verify the binding site, a ChIP assay was performed with PCR primers flanking the region of the FUS binding site and found that FUS could specifically bind to EDA2R promoter region (Fig. 4G). To further verify whether EDA2R gene was the direct target of FUS, a dual-luciferase reporter system was used. A firefly luciferase reporter construct containing the wild-type (WT) or mutant (Mut) EDA2R promoter was co-transfected with FUS overexpression plasmid or empty plasmid (Fig. 4H-I). Luciferase reporter assays showed that ectopic expression of FUS significantly enhanced the luciferase activity of the WT EDA2R promoter reporter, whereas mutation of FUS binding site abolished this effect (Fig. 4J).

To further confirm whether circHIPK3 could recruit FUS on EDA2R promoter, we performed ChIP assay in HPC with or without circHIPK3 knockdown. ChIP analysis revealed that binding of FUS to the EDA2R promoter was strikingly enhanced by HG treatment, while this association was disturbed by circHIPK3 silencing (Fig. 4K). Furthermore, circHIPK3 knockdown reduced HG-induced upregulation of EDA2R at both mRNA and protein levels (Fig. 4L-N). These data suggested that circHIPK3 facilitated the expression of EDA2R and subsequent apoptosis by recruiting FUS to the promoter of EDA2R in HPC.

### Knockdown of circHIPK3 in mouse podocyte attenuates HG-induced apoptosis through regulating EDA2R expression

**Figure 5:**
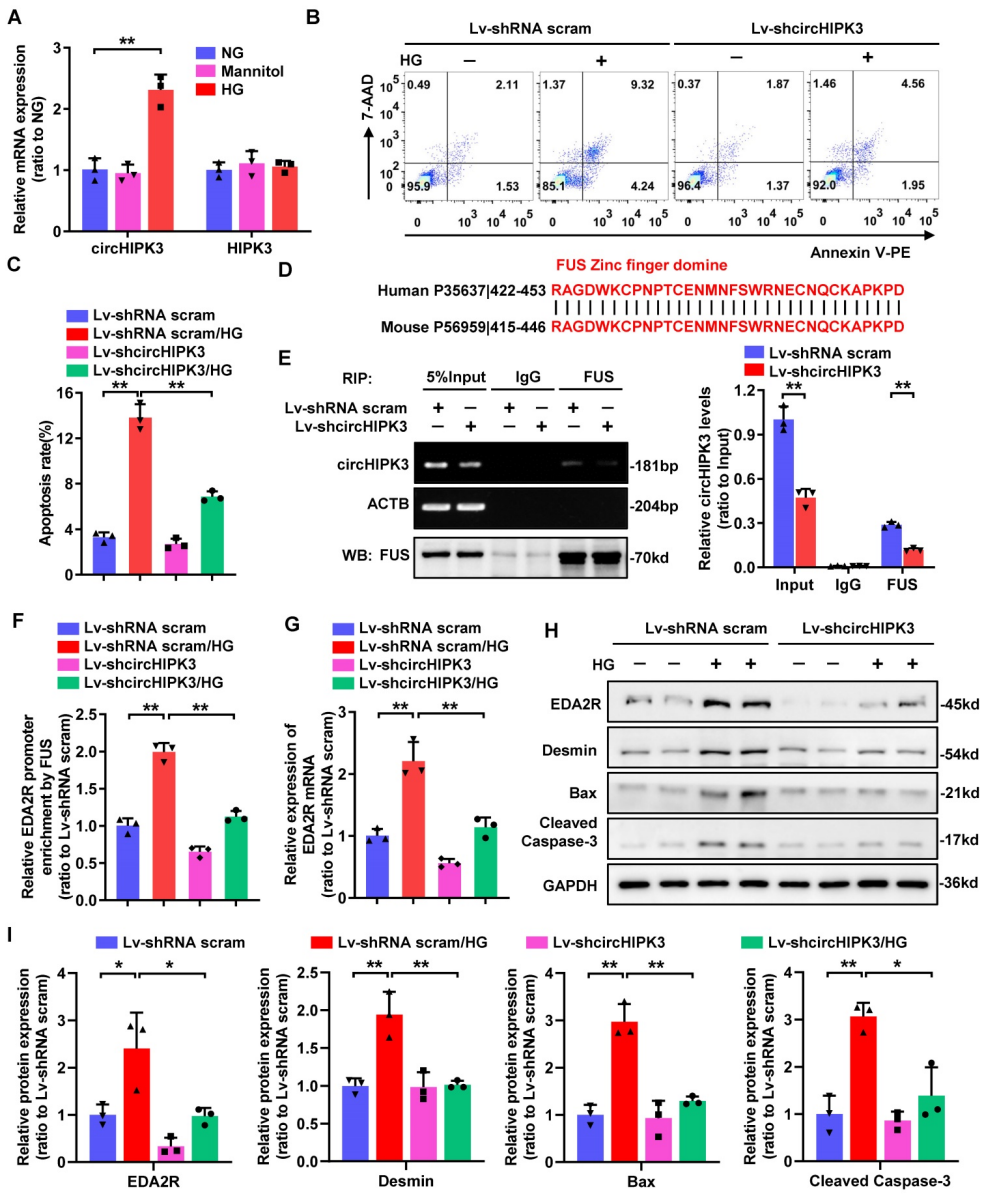
** Knockdown of circHIPK3 in mouse podocyte attenuates HG-induced apoptosis through regulating EDA2R expression. A** Expression levels of circHIPK3 and HIPK3 in MPC-5 cells cultured in media containing 5.5 mmol/L glucose (NG), 40 mmol/L glucose (HG), or mannitol/glucose (34.5 mmol/L mannitol plus 5.5 mmol/L glucose) conditions for 48h. **B, C** The effects of circHIPK3 knockdown on apoptosis in MPC-5 cells induced by HG treatment, and the quantification data (n=3). **D** The amino acid sequence of the highly conserved zinc finger (ZnF) domine displaying 100% identity between human and mouse. **E** RIP (left) and qRT-PCR (right) assays using FUS antibody showing the interaction between circHIPK3 and FUS protein in MPC-5 cells transfected with lentivirus carrying circHIPK3 shRNA or shRNA scram. The IgG-bound RNA was taken as a negative control. **F** ChIP analysis showing the interaction between EDA2R gene promoter and FUS protein in MPC-5 cells with different treatments. **G** qRT-PCR showing the expression of EDA2R in MPC-5 cells transfected with Lv-shcircHIPK3 or Lv-shRNA scram under HG (40mmol/L) treatment. Expression values were normalized to ACTB mRNA (n = 3). **H, I** Representative Western blot gel documents showing the relative protein levels of EDA2R, Desmin, Bax, and Cleaved Caspase-3 in MPC-5 cells with different treatments (n=3). Data are expressed as mean ± SD of three independent experiments. One-way ANOVA was used for comparison among multiple groups. *P < 0.05, **P < 0.01.

As circHIPK3 is highly conserved between human and mouse, we therefore evaluated whether circHIPK3 is involved in HG-induced mouse podocyte (MPC-5) injury. To delineate the role of circHIPK3 in HG-stimulated MPC-5, we firstly examined its expression pattern. Consistently, circHIPK3 expression was significantly up-regulated in HG-stimulated MPC-5 (Fig. 5A). Further studies revealed that silencing of circHIPK3 attenuated HG-induced MPC-5 apoptosis (Fig. 5B-C), indicating that circHIPK3 is indeed involved in HG-induced mouse podocyte injury. In accordance with the reported that FUS preferential binding to the “UGGU” consensus 31, we identified four conserved potential FUS-binding regions in both human and mouse circHIPK3 sequences (Supplementary Fig. 1C). Moreover, alignment of FUS protein sequence of human and mouse showing the ZnF domain is highly conserved (100% sequence identity) (Fig. 5D), indicating a possible binding between FUS and circHIPK3 in mouse podocyte. A RIP assay confirmed that endogenous FUS could interact with circHIPK3 in MPC-5, which was significantly decreased by silencing of circHIPK3 (Fig. 5E). In addition, circHIPK3 was also confirmed to recruit FUS to EDA2R promoter in MPC-5 with HG treatment (Fig. 5F). Consistently, knockdown of circHIPK3 significantly decreased HG-induced upregulation of EDA2R mRNA and protein levels, as well as increased Cleaved Caspase-3, Bax, and Desmin protein expression (Fig. 5G-I). Altogether, these results demonstrated that knockdown of circHIPK3 alleviated HG-induced MPC-5 injury via decreasing EDA2R expression, which further confirmed that circHIPK3 was a pro-apoptotic factor in podocyte.

### CircHIPK3 knockdown attenuates podocyte injury and DKD in STZ-induced diabetic mice

**Figure 6:**
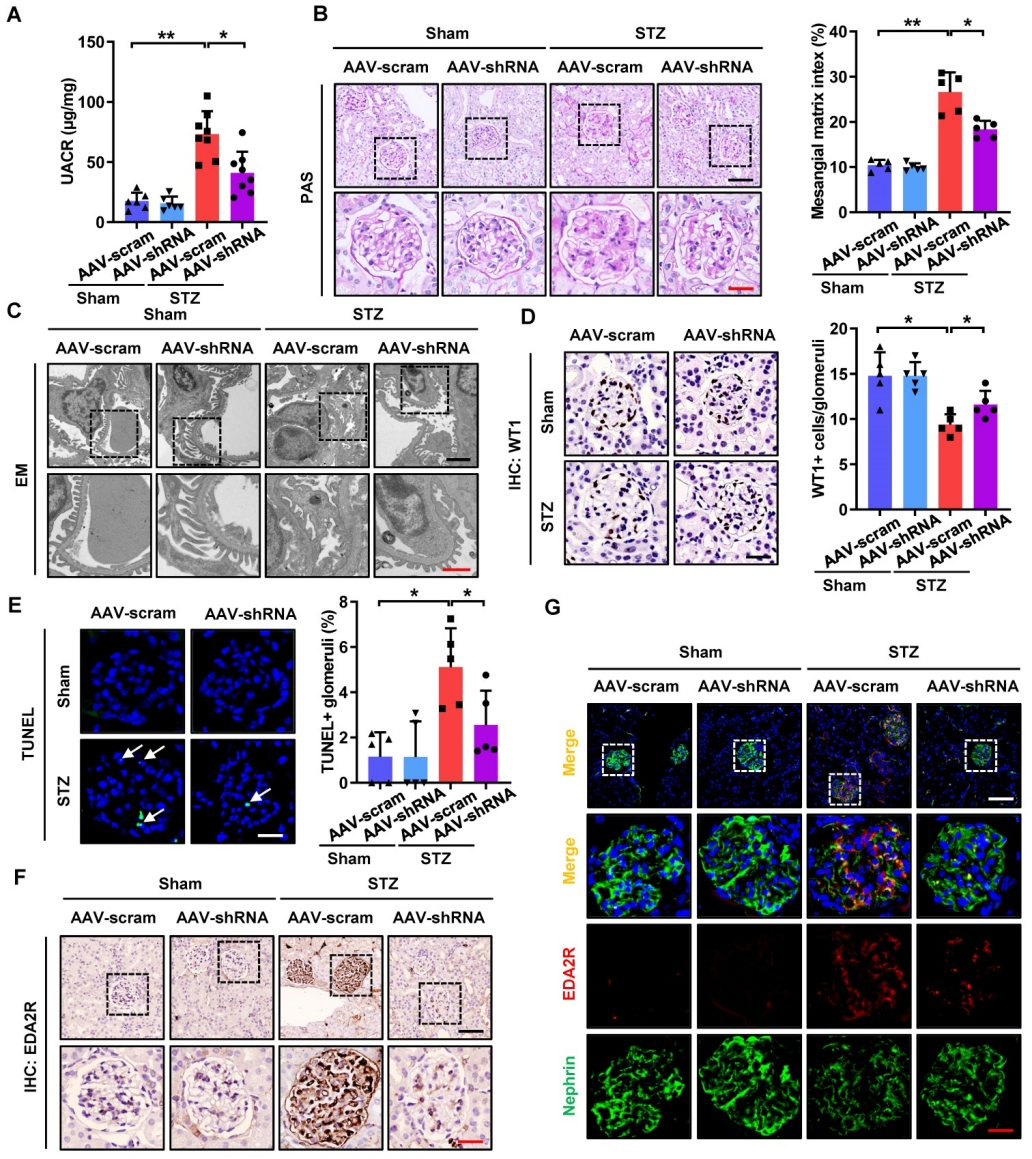
***In vivo* knockdown of circHIPK3 ameliorates renal injury in STZ-induced diabetic mice. A** Urinary albumin-to-creatinine ratio (UACR) in different groups of mice was measured. **B** Representative images of Periodic-acid-Schiff (PAS) staining and mesangial matrix index quantifications (calculated by Image J software) showing typical changes in glomerular structure in different groups of mice. Scale bar: black 50 μm, red 20 μm. **C** Representative electron microscopy analysis of renal sections in different groups of mice. Scale bar: black 2 μm, red 1 μm. **D** Representative images of WT1 IHC staining and quantification of WT1-positive glomerular cells showed that knockdown of circHIPK3 restored podocyte loss. Scale bar: 20 μm. **E** Representative images of TUNEL staining (left panel) and quantification of TUNEL-positive glomeruli (right panel) in renal sections in different groups. Scale bar: 20 μm. White arrow: TUNEL staining of positive cells. **F** IHC analysis of EDA2R expression in renal tissues in different groups of mice. Scale bar: black 50 μm, red 20 μm. **G** Representative confocal microscopic images showing the expressions of Nephrin and EDA2R in podocytes of the kidney from different groups of mice. Scale bar: white 50 μm, red 20 μm. Data are expressed as means ± SD. One-way ANOVA was used for comparison among multiple groups (n = 5-8 for each group). *P < 0.05, **P < 0.01.

As silencing of circHIPK3 significantly ameliorated HG-induced podocyte injury *in vitro*, we next examined the role of circHIPK3 in podocyte injury* in vivo*. To explore the potential for therapeutic targeting of circHIPK3, we silenced circHIPK3 in STZ-induced diabetic mice with adeno-associated virus (AAV) coated with shRNA-circHIPK3-mCherry (Supplementary Fig. 3A). Our results confirmed the efficiency of circHIPK3 knockdown *in vivo*, showing that diabetic mice received AAV-shRNA-circHIPK3 (AAV-shRNA) markedly decreased the level of circHIPK3 in isolated glomeruli by qRT-PCR analysis (Supplementary Fig. 3B). Additionally, as shown in Supplementary Fig. 3C, knockdown of circHIPK3 had no significant effects on body weight gain or the changes in blood glucose levels in the diabetic mice, which is consistent with reports from previous studies 14, 36. To examine the outcome of circHIPK3 depletion in DKD, we further evaluated renal function, urine protein levels, and histological changes in the mice. Our data demonstrated that knockdown of circHIPK3 significantly ameliorated renal injury in STZ-induced diabetic mice as evidenced by reduced albuminuria (Fig. 6A), decreased mesangial expansion (Fig. 6B), as well as attenuated podocyte foot process effacement (Fig. 6C) and podocyte loss (Fig. 6D). To further quantitatively evaluate glomerular cell apoptosis, apoptotic cells accessed by TUNEL staining were evaluated. The results showed that knockdown of circHIPK3 decreased glomerular cell apoptosis in diabetic mice (Fig. 6E). Consistently, depletion of circHIPK3 decreased the protein levels of Cleaved Caspase-3 in glomerular tissues of diabetic mice (Supplementary Fig. 3E-F). Moreover, qRT-PCR analysis (Supplementary Fig. 3D), Western blotting (Supplementary Fig. 3E-F), IHC staining (Fig. 6F), and immunofluorescence (Fig. 6G) results further confirmed that silencing circHIPK3 significantly decreased EDA2R expression and restored the loss of podocyte marker in STZ-induced diabetic mice. Taken together, these data suggested that knockdown of circHIPK3 expression remarkably attenuated podocytes injury and DKD progression in diabetic mice.

## Discussion

**Figure 7:**
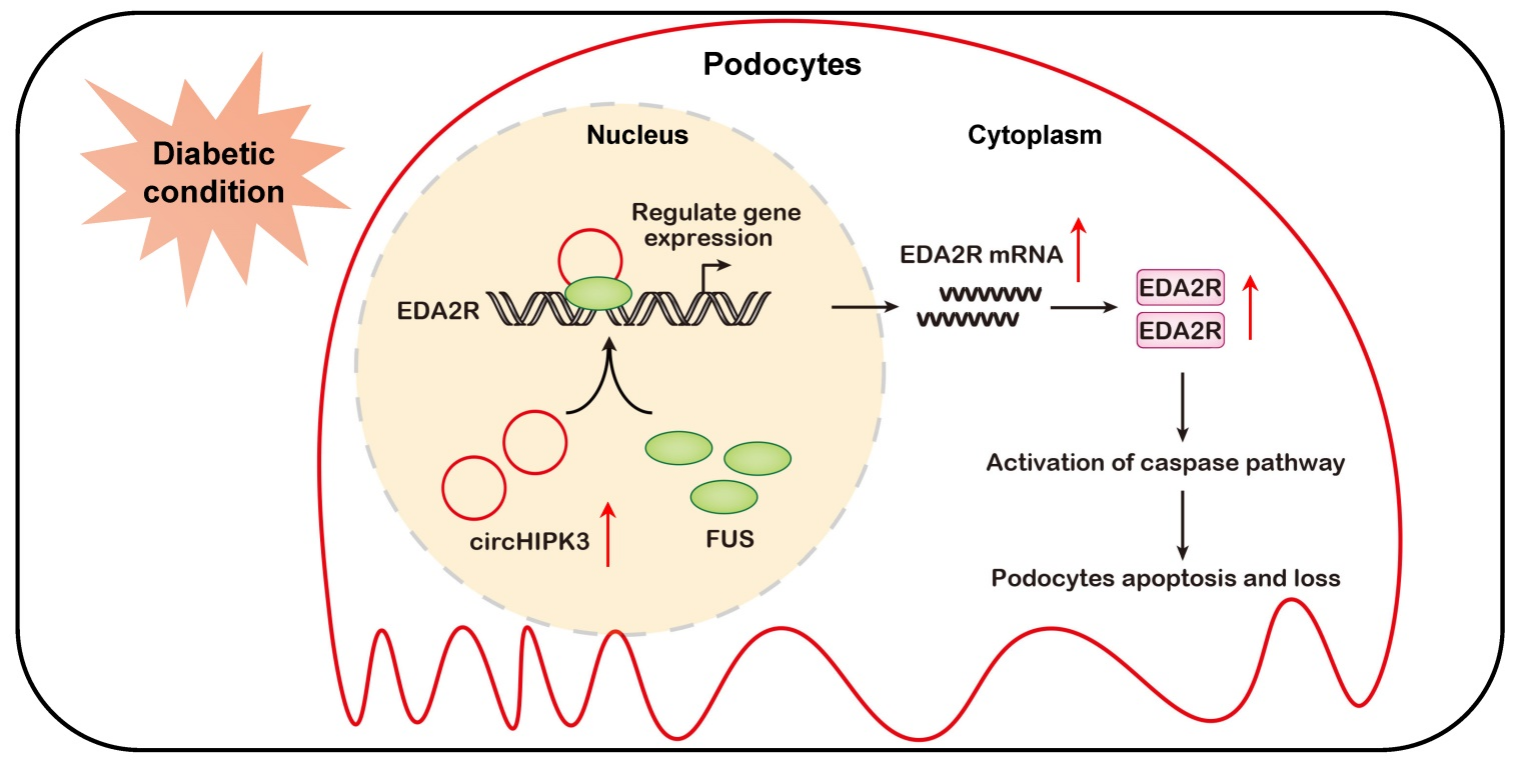
** Schematic depicting circHIPK3 upregulation exacerbates podocyte injuries and proteinuria in DKD through facilitating transcriptional activation of EDA2R in a FUS dependent manner.** Under diabetic conditions, upregulated circHIPK3 facilitates the enrichment of FUS on EDA2R promoter, resulting in activation of EDA2R-mediated apoptosis signaling pathway in podocytes.

In the present study, we identify circHIPK3, a highly conserved circRNA, as an important signature circRNA in DKD. Our evidence shows that the level of circHIPK3 is increased in podocyte cultured in HG condition and diabetic mice glomerular, and silencing circHIPK3 by adeno-associated virus-mediated RNA interference ameliorates podocyte injury and albuminuria in STZ-induced diabetic mice. Mechanistically, circHIPK3 promotes the enrichment of FUS on EDA2R promoter, resulting in the upregulation of EDA2R expression and activation of apoptotic signaling (Fig. 7). These findings highlight a novel role for circHIPK3 in regulating podocyte injury in DKD, representing a promising therapeutic target for DKD.

CircRNAs function as important regulators in various physiological and pathophysiological process in human health and disease 11. Emerging studies have demonstrated that circRNAs located in cytoplasmic can function as efficient miRNA sponges to inhibit miRNA-target interactions 10-12. Alternatively, circRNAs can also facilitate chromatin modifications and promote gene expression in the nucleus. For example, circ-CTNNB1 accelerates tumor progression through DDX3 mediated transactivation of YY1 23. In addition, ci-ankrd52 regulates target gene expression by modulating the elongation activity of RNA polymerase II 37. Previous studies have shown that circHIPK3 mainly function as an endogenous miRNA sponge to regulate the expression of target genes 14, 36, 38, 39. In our study, we expand the understanding of the mechanisms of circHIPK3 by showing that circHIPK3 can also function via FUS-mediated target gene transcriptional activation.

As a member of the ten-eleven translocation (TET) family, FUS is predominantly expressed in nucleus and involved in numerous cellular processes, including the regulation of transcription and translation 40. FUS has been identified as a vital modulator of cell apoptosis. In prostate cancer, increased expression of FUS promotes cell death through activation of apoptotic pathways 41. Meanwhile, ectopic expression of FUS induces neurotoxicity through the mitochondrial apoptotic pathway 42. While on the contrary, silencing of FUS activates cell apoptosis in gastric cancer 43. These findings indicate that FUS exerts pro-apoptotic or anti-apoptotic effects in a context-dependent manner. As a transcription factor, FUS has been reported to bind to the promoters of target genes such as COL4A2 35, CCND1 41, and MECP2 44.

Recent studies also reveal that circFndc3b plays a protective role in myocardial infarction via interacting with FUS to regulate VEGF expression 45. However, the role of circRNA in regulating FUS activity in DKD progression remains to be determined. In the current study, our results indicated that circHIPK3 could directly bind to the ZnF domain of FUS protein. We speculate that the binding of circHIPK3 may result in altered FUS protein structure that facilitates its binding to the target gene promoter, with the underlying mechanisms warranting further investigation. Because inhibition of FUS expression abolishes circHIPK3-induced podocyte apoptosis, it is suggested that the pro-apoptotic role of circHIPK3 in podocyte injury under diabetic condition is mediated, at least in part, through interacting with FUS.

RNA-seq and bioinformatics analysis were conducted to screen the downstream target genes of circHIPK3/FUS complex and further indicated that EDA2R expression was regulated by circHIPK3/FUS and mediated circHIPK3-induced podocyte injury in DKD. EDA2R, also known as TNF-RSF27, belongs the tumor necrosis factor receptor superfamily and participates in a variety of signaling pathways including cell apoptosis, immune response, inflammation, and so on 46, 47. Although EDA2R lacks a discernible death domain, it can induce apoptotic signaling through the activation of the caspase cascade and induction of apoptosis 47-49. The expression of EDA2R has been reported to be significantly up-regulated in diabetic kidneys (in both type 1 and type 2 diabetic mice) 50, 51. Moreover, another recent study reveals that EDA2R expression is up-regulated in HG-stimulated podocytes, which mediates podocyte injury through ROS generation 52. However, the specific mechanisms through which transcriptional regulators modulate EDA2R expression remains unclear. In the current study, mining of public DKD datasets reveals that higher expression of EDA2R is highly associated with apoptosis. Functional studies show that knockdown of EDA2R significantly abolishes circHIPK3-induced podocyte apoptosis, suggesting the pro-apoptotic role of EDA2R in podocyte. Additionally, our results indicate that circHIPK3 directly binds the ZnF domain of FUS to increase its binding to EDA2R promoter, resulting in transcription activation of EDA2R, which provides further evidences for the transcriptional regulation of EDA2R. Furthermore, *in vivo* knockdown of circHIPK3 by tail vein injection of AAV-shRNA significantly attenuates the upregulation of EDA2R expression in the glomeruli induced by diabetic conditions, indicating that this signaling pathway is likely to be glomerular cell-specific. Nevertheless, the underlying mechanisms warrants further investigation. Besides, it's also proved that circHIPK3 can regulate CORO1A expression, however, CORO1A does not play a major role in circHIPK3-mediated podocyte apoptosis *in vitro* (Supplementary Figure 2D-E). Several previous studies have shown that CORO1A is a member of an evolutionary conserved actin-associated family of proteins, which were initially identified as important regulators of actin cytoskeleton-dependent processes 53-55, including phagocytosis, cell migration, polarization and cytokinesis. Based on the above results and previous studies, we speculate that CORO1A may be involved in regulating circHIPK3-induced podocyte injury through other ways in DKD, whereas the precise mechanism is still worth further exploring and analyzing.

In summary, to the best of our knowledge, we have demonstrated for the first time, that circHIPK3 is up-regulated in diabetic kidneys and mediates podocyte injury and proteinuria. Mechanistic studies reveal that circHIPK3 cooperates with FUS to promote the podocyte injury and the development of DKD through facilitating transcriptional activation of EDA2R (Fig. 7). Administration of AAV-mediated shRNA targeting circHIPK3 decreases proteinuria, and prevents podocyte damage in DKD. This study extends our knowledge about the transcription regulation of EDA2R signaling by circRNA, suggesting that targeted inhibition of circHIPK3-mediated EDA2R signaling may provide a novel approach for the treatment of DKD podocyte injury.

## Supplementary Material

Supplementary figures and tables.

## Abbreviations

DKD: Diabetic kidney disease
circRNA: circular RNA
ncRNA: noncoding RNA
HG: high glucose
FUS: Fused in sarcoma
IGF2BP1/2/3: insulin-like growth factor 2 mRNA-binding protein 1/2/3
EDA2R: ectodysplasin A2 receptor
CORO1A: coronin 1A
HPC: Immortalized human podocyte
MPC: conditionally immortalized mouse podocyte
ChIP: chromatin immunoprecipitation
AAV: Adeno-associated virus
