# Supplementary material for: Regulation of Podocyte Injury by CircHIPK3/FUS Complex in Diabetic Kidney Disease
Source: Int J Biol Sci. 2022 Sep 1;18(15):5624–40. doi: 10.7150/ijbs.75994 (PMC9576511; doi:10.7150/ijbs.75994)
Supplement: Supplementary file 1 — Supplementary figures and tables. [file ijbsv18p5624s1.pdf]

Supplementary Figures and Tables

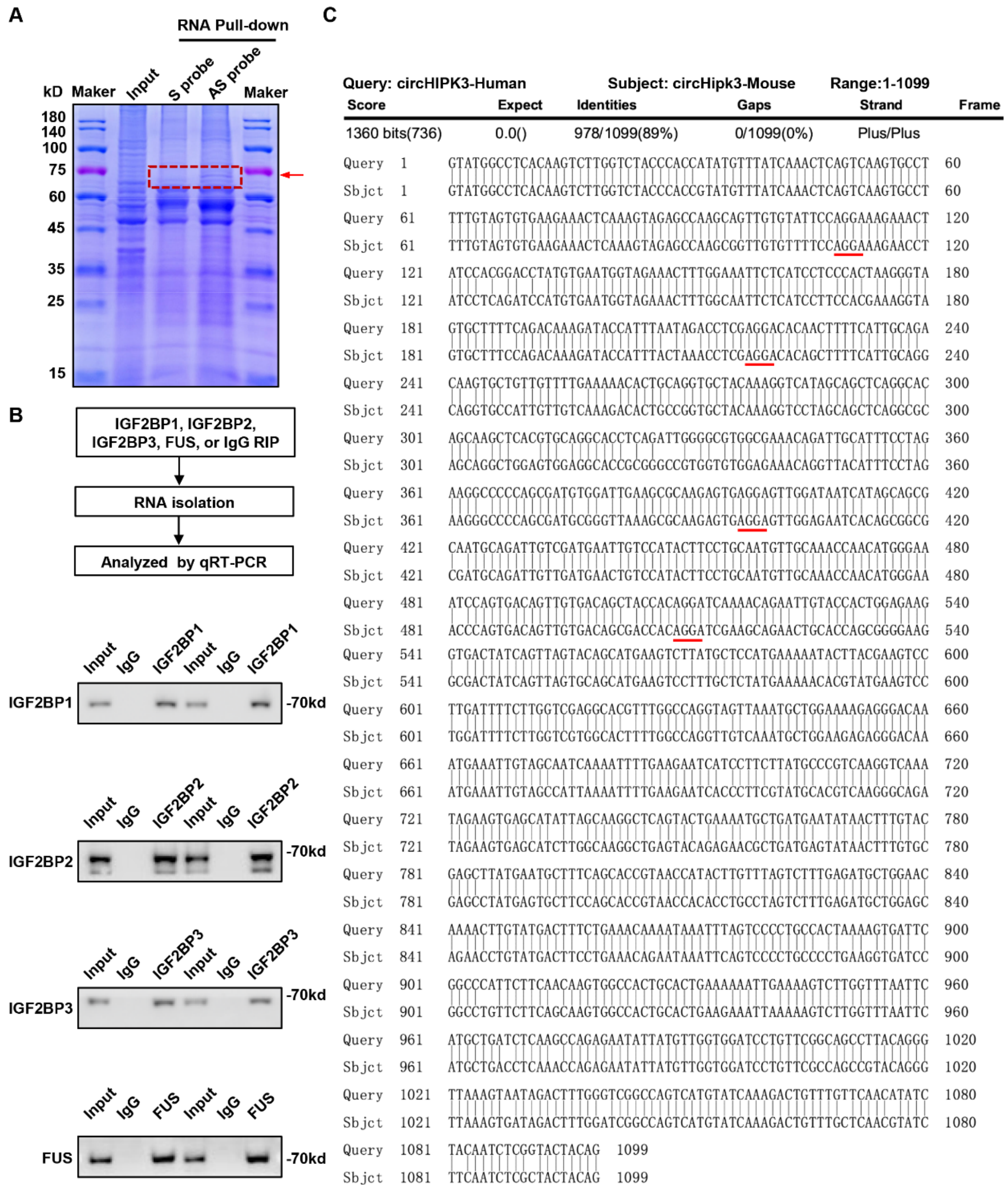

Supplementary Figure 1. Identification of circHIPK3 binding protein in HPC. A Biotin-labeled sense or antisense circHIPK3 probes were used for RNA-protein pull-down against HPC cells lysates. Identification of proteins that interact with circHIPK3 by Coomassie brilliant blue staining. Red arrow indicates the major differential band precipitated in HPC cells lysates. **B** RNA immunoprecipitation (RIP) assays in HPC

cells using IGF2BP1, IGF2BP2, IGF2BP3, FUS, and IgG antibody. The precipitate was subjected to western blotting with the antibodies against IGF2BP1, IGF2BP2, IGF2BP3, and FUS. **C** The highly conserved sequence of circHIPK3 and four conserved potential FUS-binding regions in circHIPK3 sequence between human (Query) and mouse (Subject), as indicated by red underline.

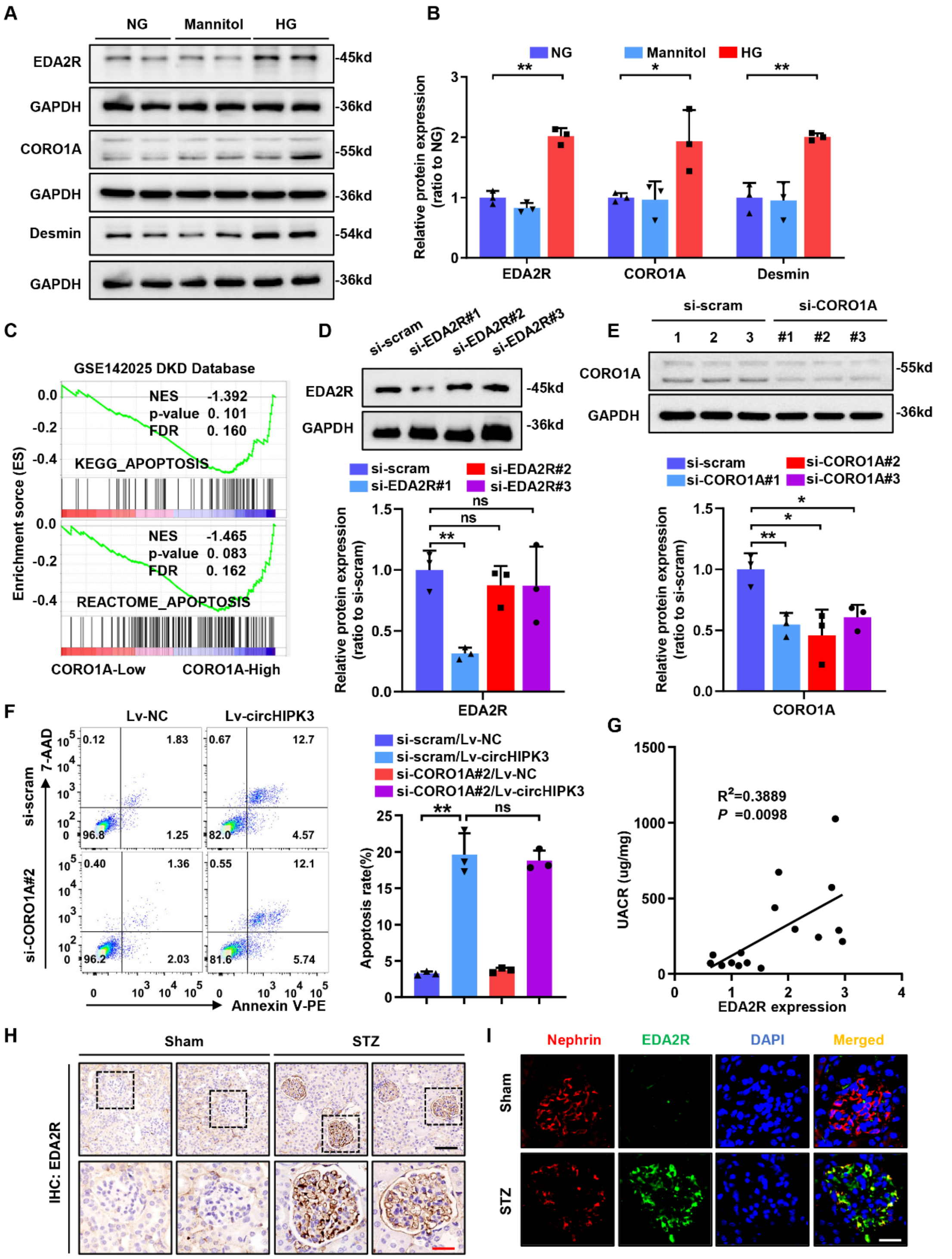

Supplementary Figure 2. EDA2R is upregulated in circHIPK3-overexpressed podocytes and diabetic mouse glomeruli. A, B Western blot analysis of EDA2R and CORO1A expression in whole-cell extracts from HPC cells with or without HG (40mmol/L) treatment (n=3). **C** The correlation between CORO1A and apoptosis

signaling pathway in DKD was analyzed by GSEA analysis. **D** Knockdown efficiencies of EDA2R were determined by western blot analysis (n=3). **E** Knockdown efficiencies of CORO1A was determined by western blot analysis (n=3). **F** Flow cytometric analysis of apoptotic cells among HPCs with different treatments. Quantification of the apoptotic cells was showed at right panel (n=3). **G** Linear regression analysis of the EDA2R expression with urinary albumin-to-creatinine ratio (UACR) in Nephroseq database. **H** Representative images of EDA2R immunohistochemical in kidney sections. Scale bar: black 50 μm, red 20 μm. **I** Representative confocal microscopic images showing the levels of EDA2R in podocytes of the kidney from different groups of mice. Nephrin was used as a podocyte marker. Scale bar, 20 μm. Values are expressed as mean ± SD of three independent experiments. One-way ANOVA was used for comparison among multiple groups. Student's t-test was employed for comparisons between two groups. *$P < 0.05$, **$P < 0.01$, ns represents no significant.

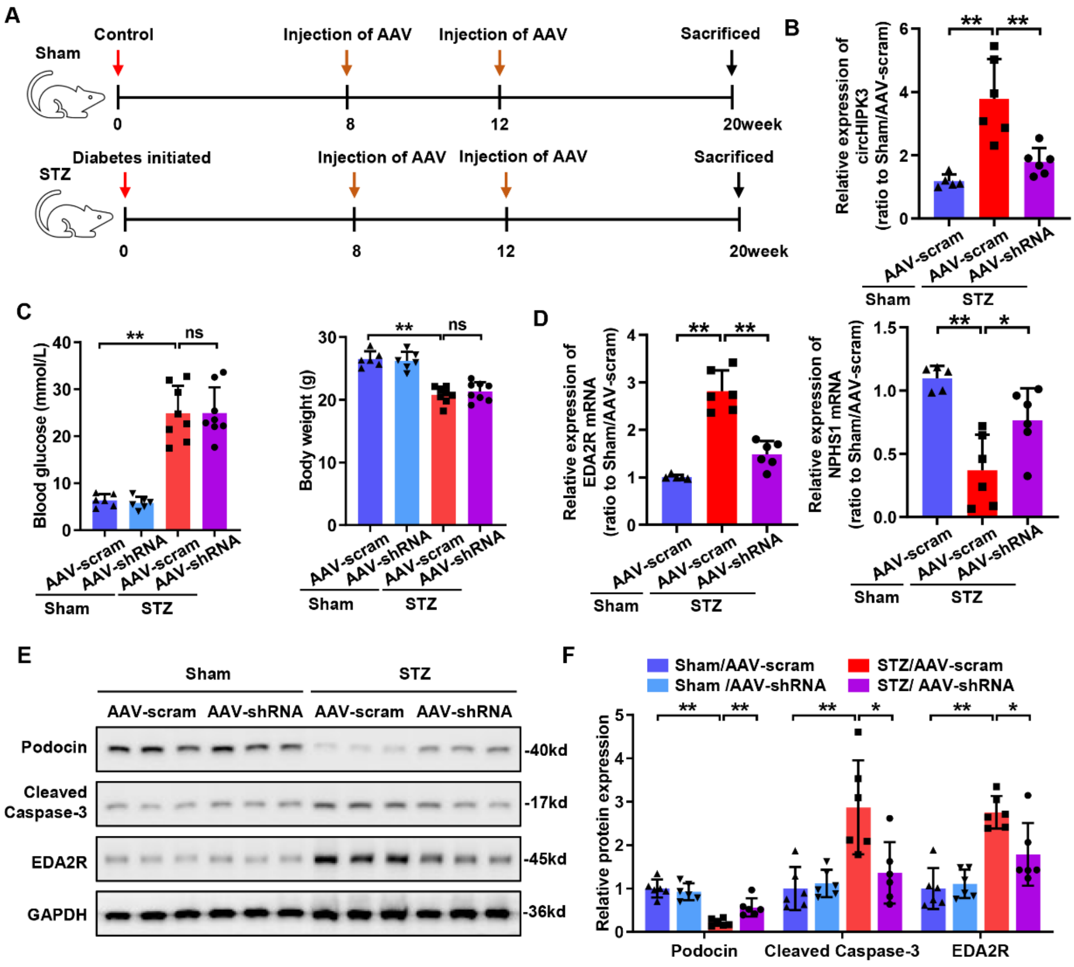

Supplementary Figure 3. Knockdown of cicHIPK3 *in vivo* attenuates renal injury in STZ-induced diabetic mice. A Schematic diagram depicting the *in vivo* experimental procedure. **B** qRT-PCR analysis showing that AAV delivery of circHIPK3 shRNA (AAV-shRNA) effectively downregulated circHIPK3 expression in isolated glomeruli in STZ-induced DKD mice. **C** Results showing that *in vivo* knockdown of circHIPK3 had no significant effect on blood glucose levels and body weight. **D** The relative mRNA levels of NPHS1 and EDA2R in isolated glomeruli in different groups were evaluated by qRT-PCR analysis. **E, F** Western blot analysis of EDA2R, Cleaved Caspase-3, and podocin expression in glomerular tissues from indicated mice (n=6). Data are expressed as mean ± SD. One-way ANOVA was used for comparison among

multiple groups (n = 5-8 for each group). *$P < 0.05$, **$P < 0.01$, ns represents no significant.

Supplementary Table 1. Primer sets used for qRT-PCR, RIP, ChIP, and plasmid construction.

Primer set	Primers	Sequence	Application
GAPDH (human)	Forward	CAGCCTCAAGATCATCAGCAAT	qRT-PCR
	Reverse	TCTTCTGGGTGGCAGTGATG	
circHIPK3 human)	Forward	GGTCGGCCAGTCATGTATCA	qRT-PCR, RIP
	Reverse	ACACAACTGCTTGGCTCTACT	
HIPK3 (human)	Forward	TGGATGATGTAGCGCATGTGA	qRT-PCR
	Reverse	AAGGATGGTTCAGGGTCTCAG	
U6 (human)	Forward	GGAACGATACAGAGAAGATTAGC	qRT-PCR
	Reverse	TGGAACGCTTCACGAATTTGCG	
FUS (human)	Forward	ACGTCATGACTCCGAACAGG	qRT-PCR
	Reverse	AATCATGGGCTGTCCCGTTT	
VXN (human)	Forward	ATCTCTGATACCGCCAGTGC	qRT-PCR
	Reverse	TTGGGGAAGGGCTGGATACT	
CERKL (human)	Forward	TCCTTAACCCCCAAAGTCACAA	qRT-PCR
	Reverse	CAATGGTTTCCGATGCCCAC	
EDA2R (human)	Forward	ATTGCCAGCGTGGAGGTTT	qRT-PCR
	Reverse	TGCAGGAGGCCATGGTAAAG	
CORO1A (human)	Forward	TGATCATGGTGTGGGACGTG	qRT-PCR
	Reverse	GACGGTCCTTCTCAGCTACG	
VWA5A (human)	Forward	TCCAGAACATTTACAGGGGACC	qRT-PCR
	Reverse	GAGGTGCCTTCTCCAATACCA	
SELPLG (human)	Forward	AGGAGATAAGATGGCTGGTGC	qRT-PCR
	Reverse	ACTCATATTCGGTGGCCTGTC	
ACTB (mouse)	Forward	ACTCCTATGTGGGTGACGAG	qRT-PCR
	Reverse	TCTTTTCACGGTTGGCCTTAG	
circHIPK3 mouse)	Forward	TAGACTTTGGATCGGCCAGT	qRT-PCR, RIP
	Reverse	CCTGGAAAACACAACCGCT	
HIPK3 (mouse)	Forward	CCGACCTGGAGTCCTTTCAC	qRT-PCR
	Reverse	GGTGGCATCACCGAGTTGTA	
EDA2R (mouse)	Forward	TGCCCAGGTTCTACCGAAAG	qRT-PCR
	Reverse	CCACTAGCAGACTTCCCACC	
EDA2R (-691/-514) human	Forward	TTCTCTGTCTCTGTCCGGTCT	ChIP
	Reverse	ACAAGGAGGGGGTTGAGCTA	
EDA2R (-663/-507) mouse	Forward	ATGTGTTCCAGGACATCCCC	ChIP
	Reverse	AGCCATCCTTTGACTTCGGT	

Oligonucleotides for FISH and RNA pull-down		
circHIPK3 (cy3)	FISH	Provided by Ribobio
U6 (cy3)	FISH	Provided by Ribobio
18s (cy3)	FISH	Provided by Ribobio
circHIPK3 Anti-sense probe	RNA pulldown	CAATCTCGGTACTACAGGTATGGCCTCACAAGTC
circHIPK3 Sense probe	RNA pulldown	GACTTGTGAGGCCATACCTGTAGTACCGAGATTG
**Primers for plasmid construction**		
Primer set	Primers	Sequence
3xFlag FUS (1-526aa)	Forward	CGCGGATCCATGGCCTCAAACGA
	Reverse	CCGCTCGAGTTAATACGGCCTCTCCC
3xFlag FUS (1-453aa)	Forward	CGCGGATCCATGGCCTCAAACGA
	Reverse	CCGCTCGAGTTAATCTGGTTTAGGGGCC
3xFlag FUS (1-371aa)	Forward	CGCGGATCCATGGCCTCAAACGA
	Reverse	CCGCTCGAGTTAGCGAGTAGCAAATGAGAC
3xFlag FUS (1-267aa)	Forward	CGCGGATCCATGGCCTCAAACGA
	Reverse	CCGCTCGAGTTAGCCACCAAATTTATTGAAGC
3xFlag FUS (268-526aa)	Forward	CGCGGATCCCCTCGGGACCAAGGAT
	Reverse	CCGCTCGAGTTAATACGGCCTCTCCC
3xFlag FUS (372-526aa)	Forward	CGCGGATCCCGGGCAGACTTTAAT
	Reverse	CCGCTCGAGTTAATACGGCCTCTCCC
3xFlag FUS (454-526aa)	Forward	CGCGGATCCGGCCCAGGAGGGGGACCA
	Reverse	CCGCTCGAGTTAATACGGCCTCTCCC
**siRNA oligo sequences**		
FUS siRNAs	Provided by Ribobio	
EDA2R siRNAs	Provided by Ribobio	
CORO1A siRNAs	Provided by Ribobio	

Supplementary Table 2. The list of proteins bound to circHIPK33 predicted by CircInteractome, CircScan, StarBase, and RBPsuite databases.

CircIntercome	CircScan	StarBase	RBPsuite
AGO1	UPF1	DDX54	AGO1
AGO2	AGO2	FAM120A	AGO2
CAPRIN1	RNPS1	FMR1	AGO3
EIF4A3	IGF2BP2	FUS	ALKBH5
FMRP	APOBEC3G	FXR1	C17ORF85
FUS	IGF2BP1	IGF2BP1	C22ORF28
HUR	CPSF2	IGF2BP2	CAPRIN1
IGF2BP1	IGF2BP3	IGF2BP3	DGCR8
IGF2BP2	FUS	LIN28B	EIF4A3
IGF2BP3	SLBP	MOV10	EWSR1
LIN28A	SND1	SRSF1	FMRP
MOV10	DDX21	TRA2A	FOX2
PTB	CPSF6	UPF1	FUS
	ELAVL1		FXR2
	PCBP2		HUR
	YWHAG		IGF2BP1
	HNRNPC		IGF2BP2
			IGF2BP3
			LIN28A
			LIN28B
			METTL3
			MOV10
			PTB
			PUM2
			SFRS1
			TIAL1
			TNRC6
			U2AF65
			WTAP
			ZC3H7B

Supplementary Table 3. The differentially expressed genes were screened in HPC treated with circHIPK3 overexpression by RNA-seq.

Gene	Lv-circHIPK3_1	Lv-circHIPK3_2	Lv-NC_1	Lv-NC_2	log2 FC	adjust *P*-value
HIPK3	14.8883	14.5319	10.0590	10.1896	4.5858	0.0000
MYH14	5.6598	5.8974	3.7673	4.6100	1.5899	0.0040
RN7SL1	13.0098	11.5275	10.5494	10.8335	1.5772	0.0281
NAT8L	6.6307	6.0493	4.2588	5.4385	1.4913	0.0217
RN7SK	14.0416	13.0725	12.1005	12.0662	1.4737	0.0079
PALM3	7.6057	7.7166	6.3720	6.1367	1.4068	0.0002
SEPSECS-AS1	6.7117	5.9729	5.1748	4.7171	1.3963	0.0066
HOXC9	12.8819	12.7523	11.6834	11.3923	1.2793	0.0004
CH507-513H4.5	18.5035	17.2391	16.3978	16.9329	1.2059	0.0498
RP11-755J8.1	6.3396	6.3071	5.0071	5.2313	1.2042	0.0003
VWA5A	5.4497	5.4912	4.0341	4.6919	1.1074	0.0065
CH507-513H4.3	14.5328	13.4065	12.5910	13.1390	1.1047	0.0499
RP11-334A14.2	9.0446	9.1998	7.4847	8.5714	1.0942	0.0349
PSMD6-AS2	7.6716	7.7415	6.6536	6.6958	1.0318	0.0003
TG	5.1318	4.9563	3.8212	4.2380	1.0145	0.0029
HNRNPA3P3	8.1871	8.5207	6.8902	7.8425	0.9875	0.0378
PDE4C	5.1451	5.3097	3.9826	4.6648	0.9037	0.0172
GRK4	6.9375	6.9050	6.2007	5.8681	0.8869	0.0024
MAP1LC3A	8.5551	8.4902	8.1327	7.1749	0.8688	0.0491
GTF2IP12	6.6611	6.0246	5.5675	5.4124	0.8529	0.0173
RAB43P1	9.4515	9.2841	8.6692	8.3881	0.8392	0.0029
RP1-121G13.3	8.5299	8.8267	8.0769	7.6192	0.8303	0.0112
RP11-46D6.1	8.5504	8.4818	7.7592	7.6150	0.8290	0.0010
AP001412.1	8.5364	8.6194	7.2984	8.2006	0.8284	0.0484
DNAJC12	8.0052	8.0670	7.4057	7.0117	0.8274	0.0049
PEX11G	8.0290	7.5056	6.6756	7.2058	0.8266	0.0281
PRR29	6.9387	6.7750	6.4402	5.6643	0.8046	0.0367
TTC32	8.1194	7.5586	7.1636	6.9283	0.7931	0.0181
ITFG1-AS1	9.7060	9.7136	8.7907	9.0464	0.7912	0.0023
NACAP1	8.4334	8.4965	7.7280	7.6772	0.7623	0.0010
TMEM60	9.6550	9.4299	8.8500	8.7249	0.7550	0.0028
PIN4P1	11.3358	11.2397	10.8713	10.1965	0.7539	0.0298
ZNF670-ZNF695	8.5075	8.1825	7.9703	7.2223	0.7487	0.0496
ICAM2	7.7067	7.7053	7.2526	6.6733	0.7430	0.0199
RP11-113K21.4	12.6555	12.9404	11.9486	12.1762	0.7356	0.0063
FTH1P2	10.1598	10.5535	9.9127	9.3418	0.7294	0.0348
DBIL5P	7.5684	7.0712	6.8772	6.3371	0.7127	0.0437
TTC28-AS1_2	12.4465	12.7286	12.0524	11.6989	0.7119	0.0121
CRADD	6.5391	7.2436	6.0572	6.3019	0.7118	0.0458

C1orf53	8.7612	9.1437	8.2012	8.2879	0.7079	0.0087
RPL34P27	10.4215	10.5762	9.9478	9.6484	0.7008	0.0064
NR1I3	8.1671	8.4543	7.7355	7.5154	0.6852	0.0082
TTC28-AS1_1	11.8102	11.9975	11.3388	11.1035	0.6827	0.0056
RP4-742C19.13	7.0043	7.4637	6.3869	6.7248	0.6781	0.0263
TCTEX1D2	10.0389	9.9983	9.4462	9.2356	0.6777	0.0033
FAM189A2	6.2326	6.3662	5.4237	5.8347	0.6702	0.0135
EDA2R	7.8353	7.8029	7.1534	7.1526	0.6661	0.0016
AC009302.2	9.4869	9.5284	8.8489	8.8360	0.6651	0.0017
SELPLG	9.1693	8.6170	8.3944	8.0660	0.6630	0.0388
KPNA5	8.5348	8.4590	7.9160	7.7607	0.6585	0.0029
AC005614.3	7.4543	7.3883	6.4959	7.0300	0.6584	0.0250
RP11-490E15.2	9.6290	9.1159	8.8346	8.5994	0.6555	0.0288
RP11-517H2.6	10.9455	10.9421	10.3313	10.2502	0.6531	0.0020
HNRNPA3P1	9.2005	9.1994	8.7621	8.3404	0.6487	0.0144
TMEM150A	7.6433	7.8035	7.0040	7.1493	0.6468	0.0041
RAD51-AS1	7.6102	7.6405	7.0976	6.8623	0.6454	0.0047
CORO1A	6.3511	6.4218	5.7088	5.7783	0.6429	0.0023
DIRAS1	6.7798	7.2948	6.1918	6.5970	0.6429	0.0449
RP11-783K16.5	10.4449	10.8776	9.7995	10.2473	0.6378	0.0405
FAM72A	8.2089	7.8111	7.5710	7.1770	0.6360	0.0311
ITGA7	6.1858	6.1302	5.6930	5.3558	0.6336	0.0098
FANCD2OS	8.9125	8.7962	8.3053	8.1403	0.6315	0.0041
RP11-252A24.3	7.3391	6.8916	6.4186	6.5577	0.6272	0.0209
LYPLAL1-AS1	8.7040	9.3139	8.5007	8.2654	0.6259	0.0485
LYPLAL1	8.5504	9.0006	8.1950	8.1129	0.6215	0.0204
IPO7P1	9.8909	10.3211	9.6459	9.3231	0.6215	0.0304
RP11-345P4.9	7.4972	7.5488	6.9228	6.8910	0.6161	0.0024
RP13-923O23.6	10.8402	10.8902	10.0790	10.4216	0.6149	0.0113
METTL7B	7.9902	8.2573	7.7742	7.2494	0.6119	0.0401
RP11-446E9.1	8.0463	8.3764	7.7665	7.4437	0.6062	0.0228
RAB30-AS1	10.3348	10.6202	9.7698	9.9786	0.6033	0.0126
RP5-1180E21.5	10.1525	10.1200	9.5998	9.4683	0.6022	0.0035
FTH1P20	9.7024	9.9056	9.1656	9.2384	0.6020	0.0055
CTD-2583A14.8	8.8653	8.6544	7.9235	8.3987	0.5988	0.0316
RPL7L1P8	10.4004	10.3994	9.8298	9.7751	0.5974	0.0027
S100A5	8.4795	8.8429	8.3010	7.8287	0.5964	0.0448
RP11-90L1.8	8.3601	8.3666	7.7189	7.8304	0.5887	0.0034
RP11-260M2.1	9.0153	9.1404	8.2941	8.6896	0.5860	0.0200
ZBTB45P1	8.0934	8.1389	8.4296	8.9737	-0.5855	0.0384
RAB39A	7.2940	7.5611	7.8917	8.1382	-0.5874	0.0148
TRIM73	6.2169	6.5669	7.0045	6.9542	-0.5874	0.0140
C8orf46	6.2966	5.9538	6.5573	6.8737	-0.5903	0.0256
CTD-3224K15.2	8.8031	8.5482	9.3136	9.2185	-0.5903	0.0083

RP11-214O1.2	9.9112	9.5914	10.4667	10.2175	-0.5908	0.0184
C5orf56	7.5989	7.4723	8.0875	8.1676	-0.5919	0.0040
RP11-752L20.3	5.9084	5.6129	6.3809	6.3262	-0.5929	0.0098
CASS4	6.6049	6.0299	6.9732	6.8521	-0.5953	0.0435
RP3-486I3.5	9.2985	8.7965	9.5112	9.7763	-0.5962	0.0398
ACSS1	4.0829	4.5865	4.9559	4.9083	-0.5974	0.0298
RP11-144F15.1	8.2581	8.1036	8.5972	8.9676	-0.6016	0.0169
SNX18P7	8.5357	8.2036	8.7701	9.1834	-0.6071	0.0317
RP11-758P17.3	9.8125	9.4286	10.1827	10.2755	-0.6085	0.0156
POSTN	6.8900	6.5680	7.4556	7.2203	-0.6090	0.0159
RP11-247L20.4	9.0898	9.0129	9.6851	9.6425	-0.6125	0.0027
RP11-566E18.1	8.6818	8.7667	9.2836	9.3962	-0.6157	0.0033
HAPLN3	6.9212	6.9377	7.3921	7.6985	-0.6158	0.0088
NUTM2A	5.7445	6.1465	6.3930	6.7342	-0.6181	0.0295
FAM86FP	8.0591	7.9860	8.7471	8.5369	-0.6195	0.0051
CMB9-22P13.1	7.1426	7.2321	7.9381	7.6777	-0.6206	0.0070
RP11-890B15.3	6.0119	6.2077	6.6626	6.8017	-0.6223	0.0056
TMEM17	6.0046	6.0791	6.8528	6.4863	-0.6277	0.0123
RP13-638C3.4	10.1687	9.8638	10.8834	10.4079	-0.6294	0.0330
RP11-548B3.3	9.8243	10.2857	10.6097	10.7676	-0.6336	0.0223
RP11-400K9.3	8.6733	8.8945	9.5525	9.2861	-0.6354	0.0099
TRAC	6.9626	7.2863	7.9585	7.5680	-0.6388	0.0239
RP11-802D6.1	8.1306	7.9950	8.5456	8.8697	-0.6448	0.0097
GBP5	5.7857	5.4371	6.4947	6.0197	-0.6458	0.0337
DRAXIN	5.2130	5.5560	5.9112	6.1508	-0.6465	0.0143
MN1	5.6094	6.2443	6.5767	6.5722	-0.6476	0.0405
RP4-555D20.2	7.7873	7.6566	8.5812	8.1620	-0.6496	0.0157
TUBB1	6.6294	6.2902	7.1873	7.0344	-0.6511	0.0106
CTD-2033D15.3	12.9647	13.2647	13.6558	13.8825	-0.6544	0.0106
RRN3P3	8.1738	8.3824	9.0000	8.8676	-0.6557	0.0047
TBC1D3	8.4939	7.8907	8.9161	8.7854	-0.6584	0.0357
TMEM130	5.9811	6.2117	6.8135	6.6969	-0.6588	0.0049
RP11-421F16.3	8.0697	8.0830	8.8317	8.6512	-0.6651	0.0030
RP11-74E22.5	10.2705	9.9995	10.9122	10.6947	-0.6685	0.0082
RP11-458D21.1	6.5372	6.0741	7.1930	6.7651	-0.6734	0.0351
RP5-1021I20.2	9.5559	9.2604	10.3465	9.8225	-0.6763	0.0306
AC003075.4	7.9510	7.8361	8.6474	8.4936	-0.6770	0.0029
CTD-2373N4.3	8.0730	7.6255	8.6071	8.4477	-0.6781	0.0164
PDE8B	5.4770	5.5117	6.0166	6.3339	-0.6809	0.0064
RPL39P	10.4188	9.8503	10.7954	10.8425	-0.6843	0.0255
KRTAP2-4	8.7924	8.4843	9.4024	9.2484	-0.6871	0.0072
FAM196B	6.7879	6.6211	7.2752	7.5120	-0.6892	0.0050
AC073254.1	6.8161	7.4426	7.9031	7.7388	-0.6916	0.0345
TBC1D3D	7.4716	7.2902	8.1640	7.9983	-0.7003	0.0036

RP4-584D14.7	7.5966	7.6283	8.3321	8.3192	-0.7133	0.0012
RNU6-1016P	11.2459	11.0141	11.6386	12.0587	-0.7187	0.0136
GOLGA8K	6.9959	7.1102	7.4034	8.1779	-0.7376	0.0466
SYP	5.8901	5.8916	6.5001	6.7633	-0.7409	0.0031
CERKL	6.2427	5.7248	6.5838	6.8772	-0.7467	0.0211
ASB2	6.4539	6.2150	7.3270	6.8446	-0.7513	0.0157
RP4-555D20.1	8.4805	8.1956	9.2531	8.9416	-0.7593	0.0079
BAALC	6.0515	5.4340	6.2989	6.7157	-0.7645	0.0364
RP11-193F5.4	10.5609	10.7610	11.7069	11.1784	-0.7817	0.0155
RYR3	3.7416	2.9591	4.3120	3.9657	-0.7886	0.0482
RP11-477D19.2	6.6801	7.0542	7.5077	7.8249	-0.7991	0.0096
CPLX1	6.3071	6.7836	7.2464	7.4839	-0.8198	0.0110
FAM86C2P	6.4360	5.7821	6.9720	6.9038	-0.8289	0.0194
PADI2	5.5848	6.1976	6.5532	6.8965	-0.8337	0.0230
SEC31B	5.6196	6.0090	6.6582	6.6587	-0.8442	0.0042
LINC01117	7.7894	8.0085	8.8816	8.6131	-0.8484	0.0031
RP11-110G21.1	6.8413	6.5093	7.5102	7.5618	-0.8607	0.0027
HYMAI	6.4648	5.5296	6.9215	6.8283	-0.8777	0.0448
ENO1P4	9.1214	8.4550	9.9088	9.4268	-0.8796	0.0304
RP11-845C23.3	7.8463	7.9837	8.7782	8.8584	-0.9033	0.0007
USP2-AS1	6.4363	5.6839	6.8239	7.1329	-0.9183	0.0254
AC025171.1	6.1095	6.4640	7.1890	7.2211	-0.9183	0.0024
MBL1P	6.4000	5.7826	7.0417	6.9833	-0.9212	0.0110
YY2	7.9015	7.5430	8.6207	8.6750	-0.9256	0.0024
PAQR6	5.9161	6.3166	7.2631	6.8434	-0.9369	0.0084
RP11-355O1.11	7.1894	6.4290	8.0715	7.4410	-0.9471	0.0407
RP4-584D14.5	8.2588	9.2852	9.7212	9.7304	-0.9538	0.0444
RP1-244F24.1	8.1598	7.7647	8.8222	9.0650	-0.9814	0.0036
NEAT1_1	12.5541	13.3581	13.6787	14.2409	-1.0037	0.0330
RP11-348P10.2	7.4694	7.2746	8.2974	8.4594	-1.0064	0.0008
AC004623.2	8.2153	8.5364	9.2403	9.5630	-1.0258	0.0029
RP11-173P15.5	7.8639	8.1209	9.0766	8.9756	-1.0337	0.0008
RP11-140H17.1	6.3053	6.5724	7.8967	7.0670	-1.0430	0.0201
AC116366.6	6.4732	6.1583	7.2352	7.5392	-1.0715	0.0021
OR7E126P	7.4442	7.6632	8.7207	8.5420	-1.0777	0.0007
PKI55	8.1568	7.6198	9.0584	8.9287	-1.1053	0.0037
SECTM1	5.4019	5.9258	6.9833	6.5636	-1.1096	0.0069
LINC00565	5.4669	6.3275	7.1440	6.8755	-1.1126	0.0178
RP11-996F15.5	8.3313	7.8394	9.1448	9.3055	-1.1398	0.0027
AC015849.19	8.0030	8.0530	9.4000	9.0628	-1.2034	0.0007
ZFHX4-AS1	6.0318	5.8474	7.0925	7.2001	-1.2067	0.0003
RP11-517B11.7	4.8917	5.8592	6.6943	6.7320	-1.3378	0.0114
RSPH4A	5.6225	6.2963	7.3538	7.3141	-1.3745	0.0029
RP11-473O4.5	7.4649	6.1429	8.2400	8.2547	-1.4434	0.0247
